# Association of tuberculosis risk with genetic polymorphisms of the immune checkpoint genes *PDCD1*, *CTLA-4*, and *TIM3*

**DOI:** 10.1371/journal.pone.0303431

**Published:** 2024-05-09

**Authors:** Chi-Wei Liu, Lawrence Shih-Hsin Wu, Chou-Jui Lin, Hsing-Chu Wu, Kuei-Chi Liu, Shih-Wei Lee

**Affiliations:** 1 Department of Internal Medicine, Taoyuan General Hospital, Ministry of Health and Welfare, Taoyuan, Taiwan; 2 Translational Medicine Center, Taoyuan General Hospital, Department of Health and Welfare, Taoyuan, Taiwan; 3 Graduate Institute of Biomedical Sciences, China Medical University, Taichung, Taiwan; 4 Center for Allergy, Immunology, and Microbiome (A.I.M.), China Medical University Hospital, Taichung, Taiwan; 5 Department of Nursing, Yuanpei University of Medical Technology, Hsinchu, Taiwan; Abu Dhabi University, UNITED ARAB EMIRATES

## Abstract

The immune checkpoint proteins were reported to involve to host resistance to *Mycobacteria tuberculosis (Mtb)*. Here, we evaluated 11 single nucleotide polymorphisms (SNPs) in *PDCD1*, *CTLA4*, and *HAVCR2* genes between participants with and without TB infection. Genomic DNA isolated from 285 patients with TB and 270 controls without TB infection were used to perform the genotyping assay. Odds ratios were used to characterize the association of 11 SNPs with TB risk. In this study, the various genotypes of the 11 SNPs did not differ significantly in frequency between the non-TB and TB groups. When patients were stratified by sex, however, men differed significantly from women in genotype frequencies at *HAVCR2* rs13170556. Odds ratios indicated that rs2227982, rs13170556, rs231775, and rs231779 were sex-specifically associated with TB risk. In addition, the combinations of rs2227982/rs13170556 GA/TC in men and the A-C-C haplotype of rs231775-rs231777-rs231779 in women were significantly associated with TB risk. Our results indicate that rs2227982 in *PDCD1* and rs13170556 in *HAVCR2* are associated with increased TB susceptibility in men and that the *CTLA4* haplotype appears protective against TB in women.

## Introduction

Tuberculosis (TB) is an ancient disease primarily caused by *Mycobacterium tuberculosis* (*Mtb*). It remains a socially significant condition and was the thirteenth leading cause of death in 2019 [1]. In recent years, associations of genetic variations in host immune-related genes and TB susceptibility have been reported [2–4], and some research has implicated immune checkpoint proteins in TB risk [5, 6]. These proteins, including programmed cell death-1 (PD-1), cytotoxic T-lymphocyte antigen-4 (CTLA-4), and T-cell immunoglobulin and mucin domain 3 (TIM3), represent the immune regulatory function of co-inhibitory receptors associated with T-cell dysfunction and exhaustion in many cancers [6–8]. Although the possible involvement of immune checkpoint proteins in *Mtb* infection has attracted increasing focus [9–11], the association of their genetic polymorphisms with TB susceptibility is not well understood. Careful examination of the relationship between these polymorphisms and TB risk would increase understanding about human susceptibility to TB.

PD-1 that is encoded by the *PDCD1* gene on human chromosome 2, which consists of 5 exons, is expressed during T-cell activation. Through its ligands–programmed cell death 1 ligand 1 (PD-L1) and PD-L2 it counters positive signals through the T-cell receptor and CD28 [12]. Patients with active TB have higher levels of CD4+ T-cell PD-1 expression compared with unaffected controls [13, 14]. In addition, PD-1^−/–^mice are more susceptible to *Mtb*-induced mortality because of excessive inflammation and uncontrolled bacterial proliferation in the lungs [15]. Also in mice, PD-1 ligation decreases interferon (IFN)-γ production by CD4+ T cells and results in exacerbated pulmonary *Mtb* infection and early host mortality [16]. CD4+ T cells and macrophages enhance phagocytosis and intracellular killing of *Mtb in vitro* though blocking the PD-1/PD-L1 pathway, suggesting an important role for PD-1 in *Mtb* infections [13].

CTLA4 is encoded by the *CTLA4* gene that is located on human chromosome 2 and consists of 4 exons. CTLA-4, a transmembrane homodimer glycoprotein, is expressed by activated effector T cells and negatively regulates T-cell immune responses [17]. Furthermore, patients with latent TB infection with *Mtb* have higher levels of PD-1, CTLA-4, and TIM3 in lymphocytes compared with unaffected controls, possibly because of an evasion of immune exhaustion in the *Mtb* infection [18]. In peripheral blood mononuclear cells from patients with latent TB infection, *Mtb* culture-filtrate antigen was shown to induce production of IFN-γ and interleukin (IL)-17 after CTLA-4 blockade [19].

TIM3, a member of the TIM family of immunoregulatory proteins, is associated with regulation of immune responses in autoimmunity and cancer [20]. TIM3 is encoded by the *HAVCR2* gene on human chromosome 5, which consists of 7 exons. Previous research has indicated increased TIM3 expression in CD8+ T cells in mice during chronic *Mtb* infection [21, 22]. In patients with *Mtb* infection, high levels of TIM3 expression are also found in CD8+ T cells [23, 24]. Work with mouse models has shown that TIM3 influences T-cell exhaustion by decreasing production of IFN-γ and tumor necrosis factor [11]. In chronically infected susceptible mice, blockade of TIM3 restores T-cell function and improves bacterial control [11]. Taken together, these findings suggest an important role for PD-1, CTLA-4, and TIM3 in the host immune response during *Mtb* infection. Despite their involvement, however, the interaction of genetic polymorphisms of *PDCD1*, *CTLA4*, and *HAVCR2* in TB susceptibility is not well known.

Associations of TB susceptibility with some single nucleotide polymorphisms (SNPs) of *PDCD1* and *CTLA4* have been reported [25–27]. In *PDCD1*, rs7568402 is linked to increased TB risk [25], and rs3087243 AA in *CTLA4* is negatively associated with severe pathology in pulmonary TB [26]. In people of Southern Han Chinese ethnicity, rs231775 AG in *CTLA4* has been associated with reduced risk for TB infection [27]. However, whether other SNPs in *PDCD1*, *CTLA4*, and *HAVCR2* are associated with TB risk has not been demonstrated. To address this gap, we investigated the association of 11 SNPs of *PDCD1*, *CTLA4*, and *HAVCR2* genes with TB risk.

## Subjects and methods

### Study population

A total of 285 individuals diagnosed with *Mtb* infection were included in the study conducted at General Taoyuan Hospital in Taoyuan, Taiwan. We gathered medical record information for outpatients from January 2022 to December 2022. The inclusion criteria were age ≥20 years and diagnosis with active TB disease (i.e., evident TB lesions on X-ray or computed tomography, or positive results of sputum smears and cultures for *Mtb*). As a control group, 270 adult participants without a history of TB disease were enrolled. To avoid the interference of some antiviral and anti-cancer drugs, patients with cancer or other immune-related diseases and viral infections (e.g., hepatitis B, hepatitis C, HIV) were excluded. The study protocol was developed according to the ethical guidelines of the 1975 Declaration of Helsinki and was approved by the Ethics Committee of Taoyuan General Hospital, Taoyuan, Taiwan (TYGH110033). All participants provided written informed consent for this study.

### DNA extraction and SNP selection

Genomic DNA was extracted from peripheral blood cells or oral swabs collected from the enrolled participants respectively using a Quick-DNA™ Miniprep Kit or a QIAamp DNA Mini Kit according to the manufacturer’s instructions. Briefly, cell pellets from 300 μl of whole blood or buccal swab were lysed using the respective lysis buffer. After centrifugation at 14,000 ×*g* for 1 min, the supernatant was applied to the respective spin column for DNA purification and washed twice with wash buffer. After sterile distilled deionized water (150 μl) was added in the spin column for a 2-min incubation at room temperature, DNA was eluted by centrifuging at 14,000 ×*g* for 2 min. The extracted genomic DNA was analyzed using agarose gel electrophoresis, quantified by spectrophotometry, and stored at -80°C until SNP genotyping (Feng Chi Biotech Corp., Taipei, Taiwan). The tSNPs within the genomic regions of *PDCD1*, *CTLA4*, and *HAVCR2*, along with the 1500 base pairs upstream, were chosen using the SeattleSNPs website (http://pga.mbt.washington.edu/education.html) based on data specific to the Han Chinese population in Beijing (HapMap-HCB). The SeattleSNPs database revealed a limited number of polymorphisms (MAF > 0) within our designated region, with four SNPs identified in *PDCD1* and three in *CTLA4*. In the *HAVCR2* region, numerous SNPs were documented and organized into four bins. From these, we opted for four SNPs in *PDCD1*, three in *CTLA4*, and four in *HAVCR2* (one from each bin) for subsequent genotyping. The characteristics of the selected SNPs were presented in S1 Table.

### Genotyping assay

Genotyping of the tag SNPs was performed using the Agena MassARRAY platform with iPLEX reagent chemistry (Agena, San Diego, CA). Briefly, after amplification by PCR of the genomic DNA regions containing the tag SNPs, a single base extension reaction was performed to generate allele-specific diagnostic products. These products were identified by their unique molecular weights, using matrix-assisted laser desorption ionization–time-of-flight mass spectrometry. After allele-specific diagnostic products were loaded onto a matrix pad of a Spectro-CHIP (Agena), the Spectro-CHIP was analyzed using a MassARRAY Analyzer 4, and results were evaluated using clustering analysis with TYPER 4.0 software. The specific primer sequences of PCR and unextended primers used in this study are shown in S2 Table.

### Statistical analysis

The intermarker linkage disequilibrium (LD) of tag SNPs in the three genes was estimated, and haplotype blocks were defined based on r^2^ and D’ values, using Haploview v.4.2. Unpaired Student’s *t*-test was used to examine differences in age between non-TB and TB groups. Logistic regression and *χ*^2^ tests were used for association analyses, and odds ratios (ORs) and 95% confidence intervals (CIs) for TB risk were calculated. All statistical assessments were carried out using SPSS 21.0 software (SPSS Inc., Chicago, IL, USA).

## Results

### Participant characteristics

A total of 555 people (430 men and 125 women) were enrolled. As indicated in Table 1, we found no significant difference in sex distribution between the two groups (*p* = 0.166, *χ*^2^). The mean age of participants with TB was 57 years (range 20–92 years), and the mean age of unaffected controls was 71 years (range 22–99 years). In the overall population and within the sex-defined subgroups, the TB and non-TB groups differed significantly in age (*t*-tests).

**Table 1:** Participant characteristics.

Variables	TB, N (%)	Non-TB, N (%)	*p* value
**Gender**			
Man	214 (75)	216 (80)	0.166^a^
Woman	71 (25)	54 (20)	
**Age (years)**			
Mean ± SD (range)	57 ± 19 (20–92)	71 ± 14 (22–99)	<0.001^b^
Man, mean ± SD (range)	59 ± 18 (20–91)	74 ± 12 (33–99)	<0.001^b^
Woman, mean ± SD (range)	50 ± 20 (20–92)	60 ± 19 (22–93)	0.004^b^
**Age group-N (%)**			
< 65	184 (65)	68 (25)	<0.001^a^
≥ 65	101 (35)	202 (75)	
**Cavitation-N (%)**			
Yes	73 (26)		
No	212 (74)		
**Pleural effusion-N (%)**			
Yes	23 (8)		
No	262 (92)		

Abbreviations: SD, standard deviation; TB, tuberculosis; N, number of participants.

^a^*χ*^2^ tests

^b^*t*-tests

### Association of *PDCD1*, *CTLA4*, and *HAVCR2* polymorphisms with TB

**Fig 1:**
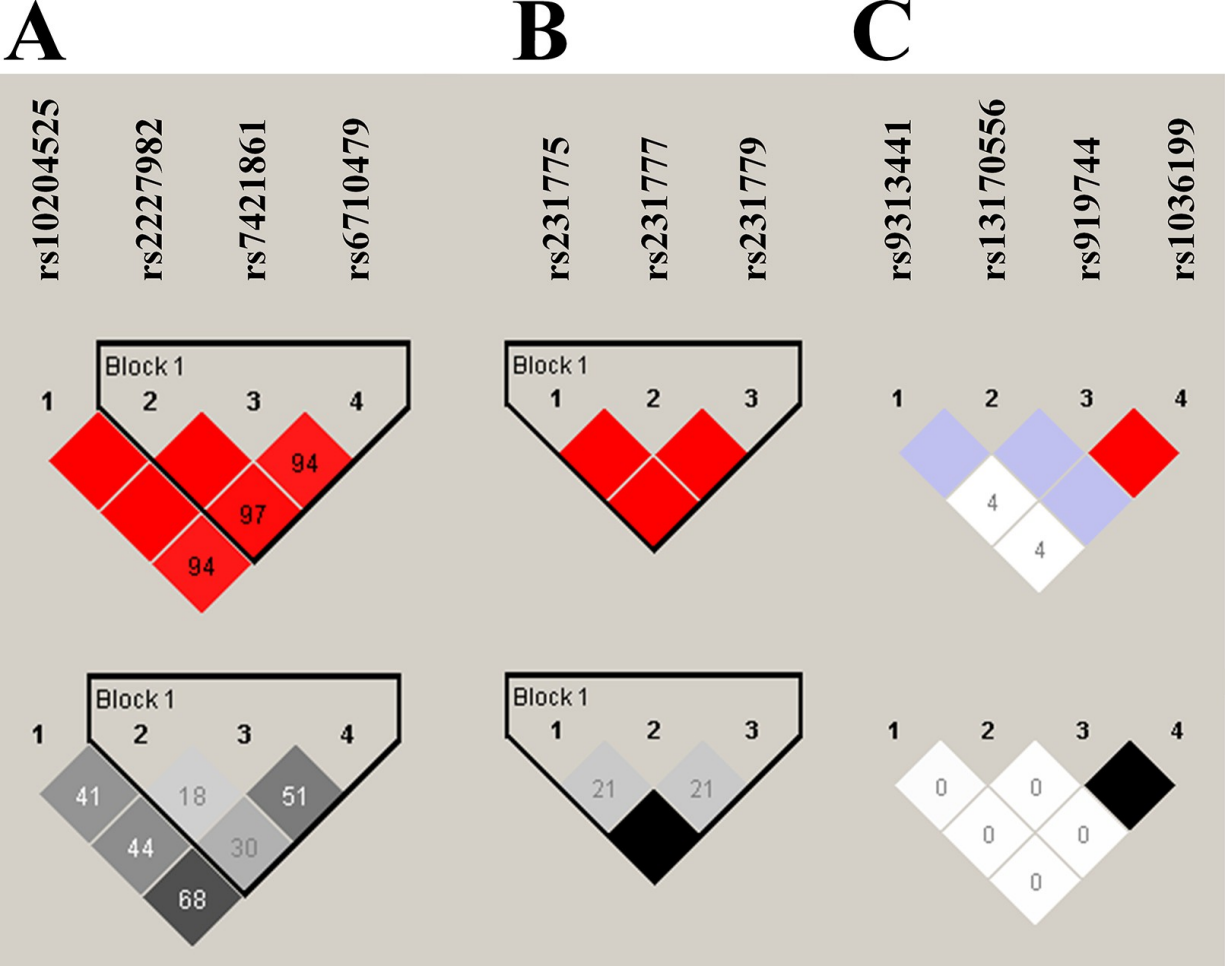
Linkage disequilibrium plot in D’ demonstrating adjacent strength between SNP pairs in *PDCD1* (10204525, rs2227982, rs7421861, and rs6710479), *CTLA4* (rs231775, rs231777, and rs231779), and *HAVCR2* (rs9313441, rs13170556, rs919744, and rs1036199). One haploblock was identified in *PDCD1* (A) and another in *CTLA4* (B).

When the 11 SNPs in *PDCD1*, *CTLA4*, and *HAVCR2* were genotyped, their genotype frequency distributions were consistent with Hardy–Weinberg equilibrium (Table 2). In the LD plot, one haploblock was identified at *PDCD1* and another at *CTLA4* (Fig 1). Selected SNPs did not differ significantly in genotype frequencies, and TB risk was not associated with these frequencies in the comparison of the non-TB and TB groups in the total population (Table 2).

**Table 2:** The differences between groups with and without TB in genotypes and alleles frequencies of selected SNPs and results of odds ratio analysis in the overall participants.

SNP	Genotype	Counts		*p* value^a^	*p*_*c*_ value	Adj. OR (95% CI)^b^	*p* value for Adj. OR
		TB group n = 285	Non-TB group n = 270				
***PDCD1***							
rs10204525	CC	25 (9)	24 (9)	0.305	NS	0.784 (0.396, 1.552)	0.485
HWp = 0.625	TC	133 (47)	109 (40)			1.262 (0.863, 1.844)	0.230
	TT (ref.)	127 (44)	137 (51)			1	
Allele model	C	183 (32)	157 (29)	0.274	NS	1.030 (0.778, 1.362)	0.839
	T (ref.)	387 (68)	383 (71)			1	
Dominant model	TT	127 (44)	137 (51)	0.145	NS	0.856 (0.596, 1.229)	0.399
	TC+CC (ref.)	158 (56)	133 (49)			1	
Recessive model	CC	25 (9)	24 (9)	0.961	NS	0.703 (0.364, 1.359)	0.294
	TT+TC (ref.)	260 (91)	246 (91)			1	
Overdominant model	TC	133 (47)	109 (40)	0.135	NS	1.307 (0.906, 1.885)	0.152
	TT+CC (ref.)	152 (53)	161 (60)			1	
rs2227982	AA	61 (21)	74 (27)	0.213	NS	0.988 (0.594, 1.642)	0.962
HWp = 0.578	GA	147 (52)	123 (46)			1.430 (0.918, 2.228)	0.114
	GG (ref.)	77 (27)	73 (27)			1	
Allele model	A	269 (47)	271 (50)	0.349	NS	0.996 (0.771, 1.287)	0.978
	G (ref.)	301 (53)	269 (50)			1	
Dominant model	GG	77 (27)	73 (27)	0.996	NS	0.794 (0.524, 1.203)	0.276
	AA+GA (ref.)	208 (73)	197 (73)			1	
Recessive model	AA	61 (21)	74 (27)	0.099	NS	0.783 (0.515, 1.190)	0.252
	GA+GG (ref.)	224 (79)	196 (73)			1	
Overdominant model	GA	147 (52)	123 (46)	0.156	NS	1.439 (0.998, 2.074)	0.051
	AA+GG (ref.)	138 (48)	147 (54)			1	
rs7421861	GG	6 (2)	8 (3)	0.462	NS	0.661 (0.202, 2.167)	0.495
HWp = 0.932	GA	85 (30)	69 (26)			1.132 (0.749, 1.711)	0.555
	AA (ref.)	194 (68)	193 (71)			1	
Allele model	G	97 (17)	85 (16)	0.566	NS	1.020 (0.718, 1.451)	0.910
	A (ref.)	473 (83)	455 (84)			1	
Dominant model	AA	194 (68)	193 (71)	0.382	NS	0.925 (0.620, 1.379)	0.702
	GA+GG (ref.)	91 (32)	77 (29)			1	
Recessive model	GG	6 (2)	8 (3)	0.520	NS	0.639 (0.196, 2.086)	0.459
	AA+GA (ref.)	279 (98)	262 (97)			1	
Overdominant model	GA	85 (30)	69 (26)	0.262	NS	1.148 (0.761, 1.732)	0.511
	AA+GG (ref.)	200 (70)	201 (74)			1	
rs6710479	CC	20 (7)	14 (5)	0.263	NS	0.961 (0.430, 2.150)	0.924
HWp = 0.834	CT	116 (41)	97 (36)			1.262 (0.862, 1.846)	0.231
	TT (ref.)	149 (52)	159 (59)			1	
Allele model	C	156 (27)	125 (23)	0.106	NS	1.119 (0.831, 1.508)	0.458
	T (ref.)	414 (73)	415 (77)			1	
Dominant model	TT	149 (52)	159 (59)	0.117	NS	0.821 (0.569, 1.183)	0.289
	CT+CC (ref.)	136 (48)	111 (41)			1	
Recessive model	CC	20 (7)	14 (5)	0.368	NS	0.876 (0.398, 1.931)	0.743
	TT+CT (ref.)	265 (93)	256 (95)			1	
Overdominant model	CT	116 (41)	97 (36)	0.248	NS	1.266 (0.871, 1.840)	0.216
	TT+CC (ref.)	169 (59)	173 (64)			1	
***CTLA4***							
rs231775	AA	31 (11)	29 (11)	0.565	NS	0.698 (0.378, 1.288)	0.250
HWp = 0.369	AG	128 (45)	133 (49)			0.729 (0.495, 1.073)	0.109
	GG (ref.)	126 (44)	108 (40)			1	
Allele model	A	190 (33)	191 (35)	0.475	NS	0.803 (0.613, 1.053)	0.113
	G (ref.)	380 (67)	349 (65)			1	
Dominant model	GG	126 (44)	108 (40)	0.315	NS	1.384 (0.957, 2.000)	0.084
	AA+AG (ref.)	159 (56)	162 (60)			1	
Recessive model	AA	31 (11)	29 (11)	0.959	NS	0.824 (0.463, 1.467)	0.510
	AG+GG (ref.)	254 (89)	241 (89)			1	
Overdominant model	AG	128 (45)	133 (49)	0.305	NS	0.785 (0.546, 1.130)	0.193
	AA+GG (ref.)	157 (55)	137 (51)			1	
rs231777	TT	1 (1)	4 (1)	0.281	NS	0.323 (0.035, 2.987)	0.319
HWp = 1.000	TC	55 (19)	45 (17)			1.169 (0.729, 1.876)	0.516
	CC (ref.)	229 (80)	221 (82)			1	
Allele model	T	57 (10)	53 (10)	0.918	NS	1.028 (0.671, 1.575)	0.899
	C (ref.)	513 (90)	487 (90)			1	
Dominant model	CC	229 (80)	221 (82)	0.652	NS	0.907 (0.572, 1.440)	0.680
	TT+TC (ref.)	56 (20)	49 (18)			1	
Recessive model	TT	1 (1)	4 (1)	0.159	NS	0.313 (0.034, 2.894)	0.306
	TC+CC (ref.)	284 (99)	266 (99)			1	
Overdominant model	TC	55 (19)	45 (17)	0.420	NS	1.184 (0.739, 1.899)	0.483
	TT+CC (ref.)	230 (81)	225 (83)			1	
rs231779	CC	31 (11)	29 (11)	0.565	NS	0.698 (0.378, 1.288)	0.250
HWp = 0.369	CT	128 (45)	133 (49)			0.729 (0.495, 1.073)	0.109
	TT (ref.)	126 (44)	108 (40)			1	
Allele model	C	190 (33)	191 (35)	0.475	NS	0.803 (0.613, 1.053)	0.113
	T (ref.)	380 (67)	349 (65)			1	
Dominant model	TT	126 (44)	108 (40)	0.315	NS	1.384 (0.957, 2.000)	0.084
	CT+CC (ref.)	159 (56)	162 (60)			1	
Recessive model	CC	31 (11)	29 (11)	0.959	NS	0.824 (0.463, 1.467)	0.510
	CT+TT (ref.)	254 (89)	241 (89)			1	
Overdominant model	CT	128 (45)	133 (49)	0.305	NS	0.785 (0.546, 1.130)	0.193
	TT+CC (ref.)	157 (55)	137 (51)			1	
***HAVCR2***							
rs9313441	AA	0	0				
HWp = 0.790	AG	22 (8)	23 (9)	0.730	NS	1.022 (0.524, 1.993)	0.948
	GG (ref.)	263 (92)	247 (91)			1	
Allele model	A	22 (4)	23 (4)	0.736	NS	1.021 (0.532, 1.962)	0.949
	G (ref.)	548 (96)	517 (96)			1	
Dominant model	GG	263 (92)	247 (91)	0.730	NS	0.978 (0.502, 1.907)	0.948
	AA+AG (ref.)	22 (8)	23 (9)			1	
Recessive model	AA	0	0	ND	ND	ND	ND
	AG+GG (ref.)	285 (100)	270 (100)				
Overdominant model	AG	22 (8)	23 (9)	0.730	NS	1.022 (0.524, 1.993)	0.948
	AA+GG (ref.)	263 (92)	247 (91)			1	
rs13170556	CC	13 (5)	8 (3)	0.055	NS	1.821 (0.671, 4.942)	0.239
HWp = 0.395	TC	91 (32)	65 (24)			1.375 (0.915, 2.068)	0.126
	TT (ref.)	181 (63)	197 (73)			1	
Allele model	C	117 (21)	81 (15)	**0.016**	**0.032**	1.379 (0.983, 1.934)	0.063
	T (ref.)	453 (79)	459 (85)			1	
Dominant model	TT	181 (63)	197 (73)	**0.017**	NS	0.704 (0.476, 1.042)	0.079
	TC+CC (ref.)	104 (37)	73 (27)			1	
Recessive model	CC	13 (5)	8 (3)	0.324	NS	1.664 (0.616, 4.493)	0.315
	TC+TT (ref.)	272 (95)	262 (97)			1	
Overdominant model	TC	91 (32)	65 (24)	**0.040**	NS	1.335 (0.891, 2.002)	0.161
	TT+CC (ref.)	194 (68)	205 (76)			1	
rs919744	GG	0	0				
HWp = 1.000	GC	9 (3)	7 (3)	0.691	NS	1.135 (0.376, 3.423)	0.823
	CC (ref.)	276 (97)	263 (97)			1	
Allele model	G	9 (2)	7 (1)	0.693	NS	1.133 (0.378, 3.390)	0.824
	C (ref.)	561 (98)	533 (99)			1	
Dominant model	CC	276 (97)	263 (97)	0.691	NS	0.881 (0.292, 2.659)	0.823
	GC+GG (ref.)	9 (3)	7 (3)			1	
Recessive model	GG	0	0	ND	ND	ND	ND
	GC+CC (ref.)	285 (100)	270 (100)				
Overdominant model	GC	9 (3)	7 (3)	0.691	NS	1.135 (0.376, 3.423)	0.823
	GG+CC (ref.)	276 (97)	263 (97)			1	
rs1036199	CC	0	0				
HWp = 1.000	CA	9 (3)	7 (3)	0.691	NS	1.135 (0.376, 3.423)	0.823
	AA (ref.)	276 (97)	263 (97)			1	
Allele model	C	9 (2)	7 (1)	0.693	NS	1.133 (0.378, 3.390)	0.824
	A (ref.)	561 (98)	533 (99)			1	
Dominant model	AA	276 (97)	263 (97)	0.691	NS	0.881 (0.292, 2.659)	0.823
	CA+CC (ref.)	9 (3)	7 (3)			1	
Recessive model	CC	0	0	ND	ND	ND	ND
	CA+AA (ref.)	285 (100)	270 (100)				
Overdominant model	CA	9 (3)	7 (3)	0.691	NS	1.135 (0.376, 3.423)	0.823
	AA+CC (ref.)	276 (97)	263 (97)			1	

Abbreviations: Ref., reference genotype; CI, confidence interval; OR, odds ratio; Pc, the Bonferroni correction of P values; HWp, p value of Hardy-Weinberg disequilibrium test.

^a^*χ*^2^ test.

^b^Adj. = adjusted for age and sex by logistic regression.

Logistic regression to evaluate interactions of sex and genotype yielded a significant interaction of sex and the rs13170556 genotype in *HAVCR2* (S3 Table). In addition, genotype and age (<65 vs ≥65 years) showed a significant interaction (S3 Table).

### Association of *PDCD1*, *CTLA4*, and *HAVCR2* polymorphisms with TB in different subgroups

The associations of *PDCD1* rs2227982, *HAVCR2* rs13170556, *CTLA4* rs231775, and *CTLA4* rs231779 with TB susceptibility were sex-dependent. Further sex-stratified analysis showed significant differences in genotype frequencies of rs13170556 between men with and without TB (Table 3). We found that male participants with the TC genotype of *HAVCR2* rs13170556 had a significantly higher risk of TB (adjusted OR [aOR] = 1.824, 95% CI = 1.134–2.935, *p* = 0.013) compared with those who had the TT genotype (Table 3). In addition, *HAVCR2* rs13170556 and TB risk were significantly associated under the allele model (aOR = 1.606, 95% CI = 1.077–2.394, *p* = 0.020), the dominant model (aOR = 0.556, 95% CI = 0.351–0.879, *p* = 0.012), and the overdominant model (aOR = 1.790, 95% CI = 1.116–2.871, *p* = 0.016). The overdominant model also showed a significant association between *PDCD1* rs2227982 and TB risk (aOR = 1.642, 95% CI = 1.070–2.519, *p* = 0.023) (Table 3).

**Table 3:** The differences between groups with and without TB in genotypes and alleles frequencies of selected SNPs and results of odds ratio analysis in male participants.

SNP	Genotype	Counts		*p* value^a^	*p*_*c*_ value	Adj. OR (95% CI)^b^	*p* value for Adj. OR
		TB group n = 214	Non-TB group n = 216				
***PDCD1***							
rs10204525	CC	17 (8)	19 (9)	0.351	NS	0.826 (0.371, 1.837)	0.639
	TC	101 (47)	87 (40)			1.358 (0.873, 2.112)	0.175
	TT (ref.)	96 (45)	110 (51)			1	
Allele model	C	135 (32)	125 (29)	0.405	NS	1.079 (0.778, 1.495)	0.649
	T (ref.)	293 (68)	307 (71)			1	
Dominant model	TT	96 (45)	110 (51)	0.208	NS	0.799 (0.524, 1.217)	0.295
	TC+CC (ref.)	118 (55)	106 (49)			1	
Recessive model	CC	17 (8)	19 (9)	0.750	NS	0.718 (0.331, 1.555)	0.401
	TT+TC (ref.)	197 (92)	197 (91)			1	
Overdominant model	TC	101 (47)	87 (40)	0.148	NS	1.395 (0.910, 2.139)	0.127
	TT+CC (ref.)	113 (53)	129 (60)			1	
rs2227982	AA	46 (22)	61 (28)	0.180	NS	0.887 (0.494, 1.591)	0.687
	GA	110 (51)	94 (44)			1.548 (0.925, 2.589)	0.096
	GG (ref.)	58 (27)	61 (28)			1	
Allele model	A	202 (47)	216 (50)	0.411	NS	0.943 (0.701, 1.270)	0.701
	G (ref.)	226 (53)	216 (50)			1	
Dominant model	GG	58 (27)	61 (28)	0.792	NS	0.789 (0.490, 1.271)	0.330
	AA+GA (ref.)	156 (73)	155 (72)			1	
Recessive model	AA	46 (22)	61 (28)	0.106	NS	0.673 (0.415, 1.093)	0.110
	GA+GG (ref.)	168 (78)	155 (72)			1	
Overdominant model	GA	110 (51)	94 (44)	0.102	NS	1.642 (1.070, 2.519)	**0.023**
	AA+GG (ref.)	104 (49)	122 (56)			1	
rs7421861	GG	4 (2)	8 (4)	0.264	NS	0.555 (0.142, 2.170)	0.397
	GA	62 (29)	51 (23)			1.397 (0.856, 2.279)	0.181
	AA (ref.)	148 (69)	157 (73)			1	
Allele model	G	70 (16)	67 (16)	0.735	NS	1.128 (0.747, 1.703)	0.567
	A (ref.)	358 (84)	365 (84)			1	
Dominant model	AA	148 (69)	157 (73)	0.421	NS	0.784 (0.490, 1.254)	0.310
	GA+GG (ref.)	66 (31)	59 (27)			1	
Recessive model	GG	4 (2)	8 (4)	0.248	NS	0.509 (0.131, 1.975)	0.329
	AA+GA (ref.)	210 (98)	208 (96)			1	
Overdominant model	GA	62 (29)	51 (23)	0.207	NS	1.427 (0.876, 2.323)	0.153
	AA+GG (ref.)	152 (71)	165 (77)			1	
rs6710479	CC	12 (5)	13 (6)	0.684	NS	0.617 (0.235, 1.623)	0.328
	CT	85 (40)	77 (36)			1.317 (0.843, 2.056)	0.226
	TT (ref.)	117 (55)	126 (58)			1	
Allele model	C	109 (25)	103 (24)	0.580	NS	1.041 (0.734, 1.476)	0.822
	T (ref.)	319 (75)	329 (76)			1	
Dominant model	TT	117 (55)	126 (58)	0.444	NS	0.836 (0.546, 1.281)	0.411
	CT+CC (ref.)	97 (45)	90 (42)			1	
Recessive model	CC	12 (5)	13 (6)	0.855	NS	0.557 (0.215, 1.441)	0.227
	TT+CT (ref.)	202 (95)	203 (94)			1	
Overdominant model	CT	85 (40)	77 (36)	0.384	NS	1.369 (0.883, 2.121)	0.161
	TT+CC (ref.)	129 (60)	139 (64)			1	
***CTLA4***							
rs231775	AA	27 (13)	24 (11)	0.838	NS	0.780 (0.392, 1.550)	0.478
	AG	96 (45)	102 (47)			0.897 (0.572, 1.405)	0.634
	GG (ref.)	91 (42)	90 (42)			1	
Allele model	A	150 (35)	150 (35)	0.920	NS	0.886 (0.647, 1.213)	0.450
	G (ref.)	278 (65)	282 (65)			1	
Dominant model	GG	91 (42)	90 (42)	0.857	NS	1.149 (0.751, 1.757)	0.522
	AA+AG (ref.)	123 (58)	126 (58)			1	
Recessive model	AA	27 (13)	24 (11)	0.629	NS	0.824 (0.431, 1.575)	0.558
	AG+GG (ref.)	187 (87)	192 (89)			1	
Overdominant model	AG	96 (45)	102 (47)	0.623	NS	0.946 (0.620, 1.445)	0.798
	AA+GG (ref.)	118 (55)	114 (53)			1	
rs231777	TT	1 (1)	4 (2)	0.315	NS	0.340 (0.036, 3.196)	0.345
	TC	43 (20)	37 (17)			1.230 (0.718, 2.104)	0.451
	CC (ref.)	170 (79)	175 (81)			1	
Allele model	T	45 (11)	45 (10)	0.963	NS	1.047 (0.649, 1.688)	0.852
	C (ref.)	383 (89)	387 (90)			1	
Dominant model	CC	170 (79)	175 (81)	0.681	NS	0.876 (0.519, 1.477)	0.619
	TT+TC (ref.)	44 (21)	41 (19)			1	
Recessive model	TT	1 (1)	4 (2)	0.181	NS	0.327 (0.035, 3.063)	0.327
	TC+CC (ref.)	213 (99)	212 (98)			1	
Overdominant model	TC	43 (20)	37 (17)	0.430	NS	1.249 (0.730, 2.136)	0.418
	TT+CC (ref.)	171 (80)	179 (83)			1	
rs231779	CC	27 (13)	24 (11)	0.838	NS	0.780 (0.392, 1.550)	0.478
	CT	96 (45)	102 (47)			0.897 (0.572, 1.405)	0.634
	TT (ref.)	91 (42)	90 (42)			1	
Allele model	C	150 (35)	150 (35)	0.920	NS	0.886 (0.647, 1.213)	0.450
	T (ref.)	278 (65)	282 (65)			1	
Dominant model	TT	91 (42)	90 (42)	0.857	NS	1.149 (0.751, 1.757)	0.522
	CT+CC (ref.)	123 (58)	126 (58)			1	
Recessive model	CC	27 (13)	24 (11)	0.629	NS	0.824 (0.431, 1.575)	0.558
	CT+TT (ref.)	187 (87)	192 (89)			1	
Overdominant model	CT	96 (45)	102 (47)	0.623	NS	0.946 (0.620, 1.445)	0.798
	TT+CC (ref.)	118 (55)	114 (53)			1	
***HAVCR2***							
rs9313441	AA	0	0			ND	ND
	AG	13 (6)	18 (8)	0.365	NS	0.655 (0.287, 1.494)	0.315
	GG (ref.)	201 (94)	198 (92)			1	
Allele model	A	13 (3)	18 (4)	0.374	NS	0.666 (0.297, 1.494)	0.324
	G (ref.)	415 (97)	414 (96)			1	
Dominant model	GG	201 (94)	198 (92)	0.365	NS	1.526 (0.669, 3.479)	0.315
	AA+AG (ref.)	13 (6)	18 (8)			1	
Recessive model	AA	0	0	ND	ND	ND	ND
	AG+GG (ref.)	214 (100)	216 (100)				
Overdominant model	AG	13 (6)	18 (8)	0.365	NS	0.655 (0.287, 1.494)	0.315
	AA+GG (ref.)	201 (94)	198 (92)			1	
rs13170556	CC	7 (3)	6 (3)	**0.010**	**0.030**	1.584 (0.452, 5.553)	0.472
	TC	74 (35)	47 (22)			1.824 (1.134, 2.935)	**0.013**
	TT (ref.)	133 (62)	163 (75)			1	
Allele model	C	88 (21)	59 (14)	**0.007**	**0.014**	1.606 (1.077, 2.394)	**0.020**
	T (ref.)	340 (79)	373 (86)			1	
Dominant model	TT	133 (62)	163 (75)	**0.003**	**0.009**	0.556 (0.351, 0.879)	**0.012**
	TC+CC (ref.)	81 (38)	53 (25)			1	
Recessive model	CC	7 (3)	6 (3)	0.765	NS	1.332 (0.381, 4.650)	0.653
	TC+TT (ref.)	207 (97)	210 (97)			1	
Overdominant model	TC	74 (35)	47 (22)	**0.003**	**0.009**	1.790 (1.116, 2.871)	**0.016**
	TT+CC (ref.)	140 (65)	169 (78)			1	
rs919744	GG	0	0			ND	ND
	GC	8 (4)	4 (2)	0.235	NS	1.430 (0.358, 5.709)	0.613
	CC (ref.)	206 (96)	212 (98)			1	
Allele model	G	8 (2)	4 (1)	0.238	NS	1.423 (0.359, 5.633)	0.615
	C (ref.)	420 (98)	428 (99)			1	
Dominant model	CC	206 (96)	212 (98)	0.235	NS	0.699 (0.175, 2.792)	0.613
	GC+GG (ref.)	8 (4)	4 (2)			1	
Recessive model	GG	0	0	ND	ND	ND	ND
	GC+CC (ref.)	214 (100)	216 (100)				
Overdominant model	GC	8 (4)	4 (2)	0.235	NS	1.430 (0.358, 5.709)	0.613
	GG+CC (ref.)	206 (96)	212 (98)			1	
rs1036199	CC	0	0			ND	ND
	CA	8 (4)	4 (2)	0.235	NS	1.430 (0.358, 5.709)	0.613
	AA (ref.)	206 (96)	212 (98)			1	
Allele model	C	8 (2)	4 (1)	0.238	NS	1.423 (0.359, 5.633)	0.615
	A (ref.)	420 (98)	428 (99)			1	
Dominant model	AA	206 (96)	212 (98)	0.235	NS	0.699 (0.175, 2.792)	0.613
	CA+CC (ref.)	8 (4)	4 (2)			1	
Recessive model	CC	0	0	ND	ND	ND	ND
	CA+AA (ref.)	214 (100)	216 (100)				
Overdominant model	CA	8 (4)	4 (2)	0.235	NS	1.430 (0.358, 5.709)	0.613
	AA+CC (ref.)	206 (96)	212 (98)			1	

Abbreviations: Ref., reference genotype; CI, confidence interval; OR, odds ratio; Pc, the Bonferroni correction of P values.

^a^*χ*^2^ test.

^b^Adj. = adjusted for age by logistic regression.

Female participants with the heterozygous *CTLA4* AG genotype of rs231775 or heterozygous CT genotype of rs231779 had significantly lower risk of TB (aOR = 0.421, 95% CI = 0.188–0.944, *p* = 0.036) compared with female participants who had the GG or TT genotype (Table 4). In addition, under the dominant model, significant associations of rs231775 and rs231779 with TB risk were observed in female population (aOR = 2.428, 95% CI = 1.107–5.323, *p* = 0.027) (Table 4).

**Table 4:** The differences between groups with and without TB in genotypes and alleles frequencies of selected SNPs and results of odds ratio analysis in female participants.

SNP	Genotype	Counts		*p* value^a^	*p*_*c*_ value	Adj. OR (95% CI)^b^	*p* value for Adj. OR
		TB group n = 71	Non-TB group n = 54				
***PDCD1***							
rs10204525	CC	8 (11)	5 (9)	0.772	NS	0.884 (0.236, 3.307)	0.854
	TC	32 (45)	22 (41)			1.123 (0.516, 2.441)	0.770
	TT (ref.)	31 (44)	27 (50)			1	
Allele model	C	48 (34)	32 (30)	0.483	NS	1.007 (0.572, 1.773)	0.980
	T (ref.)	94 (66)	76 (70)			1	
Dominant model	TT	31 (44)	27 (50)	0.482	NS	0.927 (0.441, 1.949)	0.842
	TC+CC (ref.)	40 (56)	27 (50)			1	
Recessive model	CC	8 (11)	5 (9)	0.716	NS	0.833 (0.236, 2.932)	0.775
	TT+TC (ref.)	63 (89)	49 (91)			1	
Overdominant model	TC	32 (45)	22 (41)	0.628	NS	1.147 (0.547, 2.406)	0.716
	TT+CC (ref.)	39 (55)	32 (59)			1	
rs2227982	AA	15 (21)	13 (24)	0.824	NS	1.109 (0.366, 3.362)	0.885
	GA	37 (52)	29 (54)			1.058 (0.421, 2.656)	0.905
	GG (ref.)	19 (27)	12 (22)			1	
Allele model	A	67 (47)	55 (51)	0.558	NS	1.048 (0.619, 1.774)	0.862
	G (ref.)	75 (53)	53 (49)			1	
Dominant model	GG	19 (27)	12 (22)	0.561	NS	0.933 (0.385, 2.262)	0.878
	AA+GA (ref.)	52 (73)	42 (78)			1	
Recessive model	AA	15 (21)	13 (24)	0.695	NS	1.065 (0.440, 2.579)	0.889
	GA+GG (ref.)	56 (79)	41 (76)			1	
Overdominant model	GA	37 (52)	29 (54)	0.860	NS	1.004 (0.482, 2.094)	0.991
	AA+GG (ref.)	34 (48)	25 (46)			1	
rs7421861	GG	2 (3)	0	0.462	NS	ND	ND
	GA	23 (32)	18 (33)			0.797 (0.357, 1.776)	0.578
	AA (ref.)	46 (65)	36 (67)			1	
Allele model	G	27 (19)	18 (17)	0.632	NS	0.946 (0.474, 1.889)	0.875
	A (ref.)	115 (81)	90 (83)			1	
Dominant model	AA	46 (65)	36 (67)	0.827	NS	1.185 (0.534, 2.626)	0.677
	GA+GG (ref.)	25 (35)	18 (33)			1	
Recessive model	GG	2 (3)	0	0.214	NS	ND	ND
	AA+GA (ref.)	69 (97)	54 (100)				
Overdominant model	GA	23 (32)	18 (33)	0.912	NS	0.765 (0.343, 1.703)	0.512
	AA+GG (ref.)	48 (68)	36 (67)			1	
rs6710479	CC	8 (11)	1 (2)	0.060	NS	5.608 (0.637, 49.349)	0.120
	CT	31 (44)	20 (37)			1.395 (0.647, 3.006)	0.395
	TT (ref.)	32 (45)	33 (61)			1	
Allele model	C	47 (33)	22 (20)	**0.026**	NS	1.629 (0.889, 2.986)	0.114
	T (ref.)	95 (67)	86 (80)			1	
Dominant model	TT	32 (45)	33 (61)	0.075	NS	0.626 (0.296, 1.324)	0.221
	CT+CC (ref.)	39 (55)	21 (39)			1	
Recessive model	CC	8 (11)	1 (2)	0.044	NS	4.791 (0.560, 40.988)	0.153
	TT+CT (ref.)	63 (89)	53 (98)			1	
Overdominant model	CT	31 (44)	20 (37)	0.455	NS	1.190 (0.561, 2.521)	0.651
	TT+CC (ref.)	40 (56)	34 (63)			1	
***CTLA4***							
rs231775	AA	4 (6)	5 (9)	0.189	NS	0.355 (0.080, 1.569)	0.172
	AG	32 (45)	31 (58)			0.421 (0.188, 0.944)	**0.036**
	GG (ref.)	35 (49)	18 (33)			1	
Allele model	A	40 (28)	41 (38)	0.101	NS	0.573 (0.328, 1.000)	0.050
	G (ref.)	102 (72)	67 (62)			1	
Dominant model	GG	35 (49)	18 (33)	0.074	NS	2.428 (1.107, 5.323)	**0.027**
	AA+AG (ref.)	36 (51)	36 (67)			1	
Recessive model	AA	4 (6)	5 (9)	0.437	NS	0.578 (0.143, 2.340)	0.442
	AG+GG (ref.)	67 (94)	49 (91)			1	
Overdominant model	AG	32 (45)	31 (58)	0.172	NS	0.499 (0.234, 1.067)	0.073
	AA+GG (ref.)	39 (55)	23 (42)			1	
rs231777	TT	0	0			ND	ND
	TC	12 (17)	8 (15)	0.753	NS	1.048 (0.380, 2.893)	0.928
	CC (ref.)	59 (83)	46 (85)			1	
Allele model	T	12 (8)	8 (7)	0.763	NS	1.044 (0.395, 2.756)	0.931
	C (ref.)	130 (92)	100 (93)			1	
Dominant model	CC	59 (83)	46 (85)	0.753	NS	0.954 (0.346, 2.635)	0.928
	TT+TC (ref.)	12 (17)	8 (15)			1	
Recessive model	TT	0	0	ND	ND	ND	ND
	TC+CC (ref.)	71 (100)	54 (100)				
Overdominant model	TC	12 (17)	8 (15)	0.753	NS	1.048 (0.380, 2.893)	0.928
	TT+CC (ref.)	59 (83)	46 (85)			1	
rs231779	CC	4 (6)	5 (9)	0.189	NS	0.355 (0.080, 1.569)	0.172
	CT	32 (45)	31 (58)			0.421 (0.188, 0.944)	**0.036**
	TT (ref.)	35 (49)	18 (33)			1	
Allele model	C	40 (28)	41 (38)	0.101	NS	0.573 (0.328, 1.000)	0.050
	T (ref.)	102 (72)	67 (62)			1	
Dominant model	TT	35 (49)	18 (33)	0.074	NS	2.428 (1.107, 5.323)	**0.027**
	CT+CC (ref.)	36 (51)	36 (67)			1	
Recessive model	CC	4 (6)	5 (9)	0.437	NS	0.578 (0.143, 2.340)	0.442
	CT+TT (ref.)	67 (94)	49 (91)			1	
Overdominant model	CT	32 (45)	31 (58)	0.172	NS	0.499 (0.234, 1.067)	0.073
	TT+CC (ref.)	39 (55)	23 (42)			1	
***HAVCR2***							
rs9313441	AA	0	0			ND	ND
	AG	9 (13)	5 (9)	0.548	NS	2.060 (0.601, 7.057)	0.250
	GG (ref.)	62 (87)	49 (91)			1	
Allele model	A	9 (6)	5 (5)	0.561	NS	1.942 (0.599, 6.294)	0.269
	G (ref.)	133 (94)	103 (95)			1	
Dominant model	GG	62 (87)	49 (91)	0.548	NS	0.485 (0.142, 1.663)	0.250
	AA+AG (ref.)	9 (13)	5 (9)			1	
Recessive model	AA	0	0	ND	ND	ND	ND
	AG+GG (ref.)	71 (100)	54 (100)				
Overdominant model	AG	9 (13)	5 (9)	0.548	NS	2.060 (0.601, 7.057)	0.250
	AA+GG (ref.)	62 (87)	49 (91)			1	
rs13170556	CC	6 (8)	2 (4)	0.342	NS	2.104 (0.375, 11.817)	0.398
	TC	17 (24)	18 (33)			0.559 (0.241, 1.295)	0.175
	TT (ref.)	48 (68)	34 (63)			1	
Allele model	C	29 (20)	22 (20)	0.992	NS	0.914 (0.479, 1.744)	0.784
	T (ref.)	113 (80)	86 (80)			1	
Dominant model	TT	48 (68)	34 (63)	0.588	NS	1.420 (0.651, 3.097)	0.378
	TC+CC (ref.)	23 (32)	20 (37)			1	
Recessive model	CC	6 (8)	2 (4)	0.283	NS	2.485 (0.454, 13.610)	0.294
	TC+TT (ref.)	65 (92)	52 (96)			1	
Overdominant model	TC	17 (24)	18 (33)	0.247	NS	0.527 (0.230, 1.210)	0.131
	TT+CC (ref.)	54 (76)	36 (67)			1	
rs919744	GG	0	0			ND	ND
	GC	1 (1)	3 (6)	0.192	NS	0.361 (0.035, 3.699)	0.391
	CC (ref.)	70 (99)	51 (94)			1	
Allele model	G	1 (1)	3 (3)	0.196	NS	0.370 (0.037, 3.707)	0.398
	C (ref.)	141 (99)	105 (97)			1	
Dominant model	CC	70 (99)	51 (94)	0.192	NS	2.771 (0.270, 28.400)	0.391
	GC+GG (ref.)	1 (1)	3 (6)			1	
Recessive model	GG	0	0	ND	ND	ND	ND
	GC+CC (ref.)	71 (100)	54 (100)				
Overdominant model	GC	1 (1)	3 (6)	0.192	NS	0.361 (0.035, 3.699)	0.391
	GG+CC (ref.)	70 (99)	51 (94)			1	
rs1036199	CC	0	0			ND	ND
	CA	1 (1)	3 (6)	0.192	NS	0.361 (0.035, 3.699)	0.391
	AA (ref.)	70 (99)	51 (94)			1	
Allele model	C	1 (1)	3 (3)	0.196	NS	0.370 (0.037, 3.707)	0.398
	A (ref.)	141 (99)	105 (97)			1	
Dominant model	AA	70 (99)	51 (94)	0.192	NS	2.771 (0.270, 28.400)	0.391
	CA+CC (ref.)	1 (1)	3 (6)			1	
Recessive model	CC	0	0	ND	ND	ND	ND
	CA+AA (ref.)	71 (100)	54 (100)				
Overdominant model	CA	1 (1)	3 (6)	0.192	NS	0.361 (0.035, 3.699)	0.391
	AA+CC (ref.)	70 (99)	51 (94)			1	

Abbreviations: Ref., reference genotype; CI, confidence interval; OR, odds ratio; Pc, the Bonferroni correction of P values.

^a^*χ*^2^ test.

^b^Adj. = adjusted for age by logistic regression.

We further evaluated the association of TB risk with the combination of *PDCD1* rs2227982 and *HAVCR2* rs13170556 in the male population. Male participants with the GA/TC combination had a 2.9-fold increased risk of TB (aOR = 2.926, 95% CI = 1.335–6.411, *p* = 0.007). In addition, double heterozygosity (*PDCD1*/*HAVCR2* GA/CT vs all remaining allele combinations) was still associated with a 3.1-fold increased risk of TB (aOR = 3.064, 95% CI = 1.593–5.891, *p* = 0.001) (Table 5).

**Table 5:** Combined association of TB risk and *PDCD1* rs2227982 and *HAVCR2* rs13170556 polymorphisms in male participants.

Genotype combinations	TB group n = 214	Non-TB group n = 216	*p* value^a^	*p*_*c*_ value	Adj. OR (95% CI)^a^	*p* value for Adj. OR
rs2227982/rs13170556						
GG/TT (Ref.)	40 (19)	43 (20)	**0.044**	NS	1	
GG/TC	16 (8)	16 (7)			0.727 (0.289, 1.834)	0.500
GG/CC	2 (1)	2 (1)			1.274 (0.139, 11.684)	0.830
AG/TT	67 (31)	73 (34)			1.088 (0.588, 2.014)	0.788
AG/TC	40 (19)	18 (8)			2.926 (1.335, 6.411)	**0.007**
AG/CC	3 (1)	3 (1)			1.129 (0.161, 7.927)	0.903
AA/TT	26 (12)	47 (22)			0.608 (0.297, 1.244)	0.173
AA/TC	18 (8)	13 (6)			1.427 (0.571, 3.565)	0.447
AA/CC	2 (1)	1 (1)			2.944 (0.188, 46.006)	0.441
other combinations (Ref.)	174 (81)	198 (92)	**0.002**	**0.018**	1	
AG/TC	40 (19)	18 (8)			3.064 (1.593, 5.891)	**0.001**

Abbreviations: Ref., reference genotype combination; CI, confidence interval; OR, odds ratio; Pc, the Bonferroni correction of P values.

^a^*χ*^2^-test

^b^Adjusted for age by logistic regression.

In age-stratified groups, genotype frequencies of the 11 SNPs did not differ significantly between those <65 years and those ≥65 years (S4 and S5 Tables). In addition, in these two groups, SNPs were not significantly associated with TB risk (S4 and S5 Tables).

### Association of haplotypes of *PDCD1* and *CTLA4* with TB risk

The results of LD analysis for polymorphisms of *PDCD1*, *CTLA4*, and *HAVCR2* are presented in Fig 1. Of the eight possible haplotypes, six haplotypes of *PDCD1* rs2227982-rs7421861-rs6710479 and four haplotypes of *CTLA4* rs231775-rs231777-rs231779 were detected (Table 6). In comparison with the most common haplotype of *PDCD1* (A-A-T haplotype of rs2227982-rs7421861-rs6710479) or *CTLA4* (G-C-T haplotype of rs231775-rs231777-rs231779), no haplotype was significantly associated with TB risk overall, among male participants, or in the age-stratified populations (Table 6 and S6 Table). Female participants with the A-C-C haplotype of *CTLA4* had significantly lower risk of TB (aOR = 0.488, 95% CI = 0.263–0.905, *p* = 0.023) compared with those who had the most common haplotype (G-C-T) of rs231775-rs231777-rs231779 (Table 6).

**Table 6:** Haplotype distribution of the two investigated *PDCD1* and *CTLA4* polymorphisms in the overall and sex-stratified populations.

Haplotype		Frequency (%)	TB (n)	Non-TB (n)	*p* value^a^	*p*_*c*_ value	Adj. OR (95% CI)^b^	*p* value for OR
**rs2227982-rs7421861-rs6710479**								
Total	A-A-T (ref.)	48.4	266	271	0.059	NS	1.000	
	G-A-T	25.7	142	143			0.914 (0.669, 1.248)	0.572
	G-G-C	15.7	91	84			0.973 (0.668, 1.418)	0.888
	G-A-C	9.3	62	41			1.304 (0.819, 2.075)	0.263
	G-G-T	0.6	6	1			5.646 (0.586, 54.388)	0.134
	A-A-C	0.3	3	0			ND	ND
Men	A-A-T (ref.)	48.3	199	216	0.069	NS	1.000	
	G-A-T	26.4	114	113			1.033 (0.722, 1.478)	0.860
	G-G-C	15.2	64	67			1.067 (0.687, 1.658)	0.772
	G-A-C	9.1	42	36			1.035 (0.601, 1.783)	0.900
	G-G-T	0.7	6	0			ND	ND
	A-A-C	0.3	3	0			ND	ND
Women	A-A-T (ref.)	48.8	67	55	0.059	NS	1.000	
	G-A-T	23.2	28	30			0.651 (0.338, 1.251)	0.198
	G-G-C	17.6	27	17			0.972 (0.461, 2.048)	0.940
	G-A-C	10.0	20	5			2.876 (0.992, 8.333)	0.052
	G-G-T	0.4	0	1			ND	ND
	A-A-C	0.0	0	0			ND	ND
**rs231775-rs231777-rs231779**								
Total	G-C-T (ref.)	65.7	380	349	0.618	NS	1.000	
	A-C-C	24.3	132	138			0.745 (0.548, 1.012)	0.060
	A-T-C	9.9	57	53			0.951 (0.616, 1.469)	0.821
	G-C-C	0.1	1	0			ND	ND
Men	G-C-T (ref.)	65.1	278	282	0.995	NS	1.000	
	A-C-C	24.4	105	105			0.838 (0.585, 1.200)	0.335
	A-T-C	10.5	45	45			0.999 (0.613, 1.627)	0.997
	G-C-C	0	0	0			ND	ND
Women	G-C-T (ref.)	67.6	102	67	0.163	NS	1.000	
	A-C-C	24.0	27	33			0.488 (0.263, 0.905)	**0.023**
	A-T-C	8.0	12	8			0.863 (0.321, 2.323)	0.771
	G-C-C	0.4	1	0			ND	ND

Abbreviations: Ref., reference haplotype; CI, confidence interval; OR, odds ratio; Pc, the Bonferroni correction of P values.

^a^*χ*^2^ test

^b^Adj. = adjusted for age and sex by logistic regression.

## Discussion

Previous groups have reported that some polymorphisms of *PDCD1*, *CTLA4*, and *HAVCR2* are associated with TB risk [25–28]. In this study, we found a sex-dependent association of TB risk with the AG genotype of *PDCD1* rs2227982, TC genotype of *HAVCR2* rs13170556, AG genotype of *CTLA4* rs231775, and CT genotype of *CTLA4* rs231779. In men, the AG/TC combination of *PDCD1*/*HAVCR2* was significantly associated with TB risk. In women, our haplotype analysis showed that the A-C-C haplotype of *CTLA4* rs231775-rs231777-rs231779 was associated with reduced susceptibility to TB. Our findings imply that genetic polymorphisms of *PDCD1*, *CTLA4*, and *HAVCR2* have an important role in TB susceptibility.

In men, OR analysis showed a significant association of rs2227982 AG with increased risk of TB under the additive model. *PDCD1* is located on chromosome 2, and rs2227982 (+7625 C>T) lies in the fifth exon of *PDCD1*. The rs2227982 base substitution can lead to an exchange of valine for alanine, possibly influencing the activity of PD-1 [29]. In the GTExPortal database, rs2227982 is reported to be associated with *PDCD1* expression (according to expression quantitative trait loci analysis) in subcutaneous and visceral adipose tissues (https://gtexportal.org/home/snp/rs2227982) [30]. In addition, previous studies have indicated associations of *PDCD1* rs2227982 with host susceptibility to hepatitis B infection and risk of *Mycobacterium avium* complex lung disease in women [31, 32]. A significant relationship between serum soluble PD-1 levels and different rs2227982 genotypes also has been reported [32]. PD-1 regulates *Mtb* infection and rs2227982 is associated with PD-1 expression, which may explain the association of rs2227982 with TB risk.

The distribution analyses in the current work revealed a higher frequency in the male population of the rs13170556 TC genotype among patients compared with healthy controls; thus, this genotype may play a predisposing role in TB infection. Human *HAVCR2* is located on chromosome 5, and the SNP rs13170556 lies in the fourth intron of *HAVCR2*. The OR analysis showed a sex-dependent association of the rs13170556 TC genotype with TB risk. Sex-dependent associations of rs4331426 and rs35037722 with TB risk have been reported previously, similar to studies in the Han Taiwanese population [3, 33]. The GTExPortal database indicates an association of rs13170556 with *HAVCR2* expression in testis tissue (https://gtexportal.org/home/snp/rs13170556) [30]. In addition, *HAVCR2* genetic variations have been linked to increased risk of osteoarthritis, possibly because of upregulation of IFN-γ expression by CD4+ T cells [34]. Based on these findings, we hypothesize that rs13170556 may regulate TIM3 and INF-γ expression, in turn influencing susceptibility to TB.

We also found that rs231775 AG and rs231779 CT are associated with reduced risk of TB, similar to research showing that rs231775 AG is a protective factor against TB in a Southern Han Chinese population [27]. In addition, we found that the A-C-C haplotype of the LD block (rs231775-rs231777-rs231779) in *CTLA4* is associated with reduced risk for TB in women. Some previous research has linked this LD block in *CTLA4* with immune-related diseases, such as Graves’ disease and Rasmussen syndrome [35, 36]. Human *CTLA4* is located on chromosome 2, and rs231775 (+49A>G) and rs231779 are respectively located in the first exon and intron of the *CTLA4* gene. The rs231775 base substitution at position 17 in exon 1 of *CTLA4* can lead to a change from threonine to alanine [37]. In testis tissue, the association of rs231775 and rs231779 with *CTLA4* expression has been reported in the GTExPortal database (https://gtexportal.org/home/snp/rs231775; https://gtexportal.org/home/snp/rs231779) [30]. In addition, other studies have indicated an association of rs231775 with CTLA-4 expression and an effect on the affinity of CTLA-4 for B7-1 (CD80) in many cancers [38, 39]. *CTLA4* polymorphisms also have been associated with increased secretion of cytokines such as IFN-γ and IL-2 and an enhanced immune response [40]. Taken together, the findings regarding rs231775 and rs231779 suggest an influence on susceptibility to TB through regulation of CTLA-4 expression and T-lymphocyte response.

The important effects of several host genetic factors on tuberculosis infection have been reported [41]. PD-1, CTLA4, and TIM3 were associated with host immune response against *Mtb* infection [6]. In this study, we indicated the associations of *PDCD1* rs2227982, *HAVCR2* rs13170556, *CTLA4* rs231775, and *CTLA4* rs231779 with TB risk. Though the number of subjects with different TB severity (cavitation and pleural effusion) is not sufficient to evaluate the association of selected SNPs with TB severity in our study, some previous studies have reported the association of these SNPs with clinical outcome of diseases, such as TB, primary biliary cirrhosis, and Hepatitis C [26, 42–44]. In *PDCD1* rs2227982, TB patients with TC or CC genotype have higher rate of tuberculous cavity than those with TT genotype [42]. In previous study of African Population, the *CTLA4* haplotype (rs11571315-rs733618-rs4553808-rs231774-rs231775-rs231777-rs3087243; G-A-A-A-G-C-A) of was associated with cavities in patients with TB [26]. The *CTLA4* haplotype (rs231775-rs231777-rs3087243-rs231725; G-C-G-A) was a protective factor for progression of primary biliary cirrhosis in Japanese patients [43]. Though the association between *HAVCR2* rs13170556 with clinical outcomes of TB is unknown, the protective effects of rs13170556 TC/CC genotypes with the F protein on the outcomes of HCV infection was reported [44]. In addition, the GTExPortal database indicates an association of *PDCD1* rs2227982, *HAVCR2* rs13170556, *CTLA4* rs231775, and *CTLA4* rs231779 with their gene expression in different tissues. Taken together, these finding may suggest that different genotypes in these SNPs may affect the host immune response against *Mtb* infection and clinical outcome of TB by regulating their gene expression.

The sex-dependent association of *PDCD1*, *CTLA4*, and *HAVCR2* polymorphisms with TB susceptibility were found in our study. Some previous studies also indicated that *PDCD1*, *CTLA4*, and *HAVCR2* polymorphisms were sex-dependently associated with MAC-LD risk, the resolution of hepatitis C virus infection, and the outcomes of HCV infection, respectively [32, 44, 45]. In addition, previous study indicated that the effects of some sex-specific factors on the immune response and women have a higher prevalence of autoimmune diseases compared with men [46]. The difference in function of CD4+CD25+ regulatory T-cells was observed in male and female mice [47]. Taken together, these observations may suggest that the difference in sex-dependent effect of *PDCD1*, *CTLA4*, and *HAVCR2* polymorphisms on immune response against *Mtb* infection. In our study, odds ratio analysis showed the significant associations of heterozygous genotype in *PDCD1* rs2227982, *HAVCR2* rs13170556, *CTLA4* rs231775, and *CTLA4* rs231779 with TB risk. In previous studies, the significant associations of heterozygous genotype of *PDCD1* rs2227982 and *HAVCR2* rs13170556 with the risk for breast cancer and the outcomes of HCV infection were reported, respectively [44, 48]. Though the transcriptional regulatory mechanism of different genotypes in these SNPs did not evaluate in our study, the differences in gene expression between heterozygous and homozygous genotypes of these SNPs in different tissues were found in the GTExPortal database [30]. Based on these findings, we hypothesize that the difference in gene expression between heterozygous and homozygous genotypes of these SNPs may influence immune response against *Mtb* infection. However, our findings still need more researches with large sample sizes to validate. In future, the transcriptional regulatory mechanism of these SNPs and serum levels of soluble PD-1, CTLA4, and TIM3 should be investigated.

Our study has some limitations. First, stratification by sex yielded small group sizes that could have led to an underestimation of significance. Future studies should include a larger number of participants in each group to ensure sufficient statistical power. Second, because young participants rarely have clinical evidence for the exclusion criteria, it resulted in the significant difference in age of participants between TB and non-TB groups. Third, we did not evaluate some non-genetic factors affecting susceptibility to TB, such as host status (TB contact and latent TB infection) and environment (air pollution and tobacco smoke), and thus cannot exclude the possibility of some influence of these factors in the analyses. In future, to avoid the cofounder effects, OR analysis with adjustment for non-genetic factors of subjects should be performed.

## Conclusions

We identified a significant difference in genotype frequencies of *HAVCR2* rs13170556 between men with and without TB. Our OR analysis showed sex-dependent associations of TB susceptibility and AG heterozygosity at *PDCD1* rs2227982, TC heterozygosity at *HAVCR2* rs13170556, AG heterozygosity at *CTLA4* rs231775, and CT heterozygosity at *CTLA4* rs231779. In addition, the GA/TC combination of *PDCD1* rs2227982/*HAVCR2* rs13170556 was associated with increased TB risk in men, and the A-C-C haplotype at *CTLA4* rs231775-rs231777-rs231779 was associated with reduced risk in women. Overall, *PDCD1*, *CTLA4*, and *HAVCR2* polymorphisms are sex-dependently associated with susceptibility to TB. As the TB statistics of the Taiwan CDC has been showed that men have higher TB prevalence than women [49], our results may help explain the possibility of a difference in TB prevalence between non-TB and TB subjects in men and women. However, our findings still need more researches with large sample sizes in different ethnicities to validate.

## Supporting information

S1 Table Characteristics of the selected SNPs. (DOCX)

S2 Table The sequence of primers and UEP for genotyping assay. (DOCX)

S3 Table Interaction of genetic variation and sex/age contribute to tuberculosis risk. Abbreviations: df: degree of freedom. ^a^rs231777 TT genotype is only five subjects, none in female subjects and five in male subjects above 60 years old, so the df = 4. ^b^rs1036199 CC genotype, rs9313441 AA genotype, and rs919744 GG genotype are not detected in case and control groups, so the df = 3. ^c^*p* value was calculated by logistic regression.
(DOCX)

S4 Table The differences between groups with and without TB in genotypes and alleles frequencies of selected SNPs and results of odds ratio analysis in non-aged (<65-year-old) participants. Abbreviations: Ref., reference genotype; CI, confidence interval; OR, odds ratio; Pc, the Bonferroni correction of P values. ^a^*χ*^2^ test. ^b^Adj. = adjusted for sex by logistic regression.
(DOCX)

S5 Table The differences between groups with and without TB in genotypes and alleles frequencies of selected SNPs and results of odds ratio analysis in aged (≥65-year-old) participants. Abbreviations: Ref., reference genotype; CI, confidence interval; OR, odds ratio; Pc, the Bonferroni correction of P values. ^a^*χ*^2^ test. ^b^Adj. = adjusted for sex by logistic regression.
(DOCX)

S6 Table Haplotype distribution of the two investigated *PDCD1* and *CTLA4* polymorphisms in the age-stratified populations. Abbreviations: Ref., reference genotype; CI, confidence interval; OR, odds ratio; Pc, the Bonferroni correction of P values. ^a^*χ*^2^ test. ^b^Adj. = adjusted for age and sex by logistic regression.
(DOCX)
