# Supplementary material for: Association of tuberculosis risk with genetic polymorphisms of the immune checkpoint genes PDCD1, CTLA-4, and TIM3
Source: PLoS One. 2024 May 9;19(5):e0303431. doi: 10.1371/journal.pone.0303431 (PMC11081348; doi:10.1371/journal.pone.0303431)
Supplement: S1 Table — (DOCX) [file pone.0303431.s001.docx]

| **SNP ID** | **Chr** | **Location** | **Genomic feature** |
| --- | --- | --- | --- |
| ***PDCD1*** |  |  |  |
| rs10204525 | chr2:241850169 | 3 Prime UTR | 3 Prime UTR Variant |
| rs2227982 | chr2:241851281 | Exon 5 | Missense, A [GCC] > V [GTC] |
| rs7421861 | chr2:241853198 | Intron 1 | Intron Variant |
| rs6710479 | chr2:241855866 | Intron 1 | Intron Variant |
| ***CTLA4*** |  |  |  |
| rs231775 | chr2:203867991 | Exon 1 | Missense, T [ACC] > A [GCC] |
| rs231777 | chr2:203868865 | Intron 1 | Intron Variant |
| rs231779 | chr2:203869764 | Intron 1 | Intron Variant |
| ***HAVCR2*** |  |  |  |
| rs9313441 | chr5:157089149 | Intron 5 | Intron Variant |
| rs13170556 | chr5:157095577 | Intron 4 | Intron Variant |
| rs919744 | chr5:157104243 | Intron 3 | Intron Variant |
| rs1036199 | chr5:157104725 | Exon 3 | Missense, R [CGG] > Q [CAG] |

**S1 Table. Characteristics of the selected SNPs.**
