# Supplementary material for: Association of tuberculosis risk with genetic polymorphisms of the immune checkpoint genes PDCD1, CTLA-4, and TIM3
Source: PLoS One. 2024 May 9;19(5):e0303431. doi: 10.1371/journal.pone.0303431 (PMC11081348; doi:10.1371/journal.pone.0303431)
Supplement: S2 Table — (DOCX) [file pone.0303431.s002.docx]

**S2 Table. The sequence of primers and UEP for genotyping assay.**

| **SNP** | **1st primer** | **2nd primer** | **UEP** |
| --- | --- | --- | --- |
| ***PDCD1*** |  |  |  |
| rs10204525 | ACGTTGGATGTTCAGGAATGGGTTCCAAGG | ACGTTGGATGTGTTGGGAGGGCAGAAGTG | CACCTAGGGCCCCCCAT |
| rs2227982 | ACGTTGGATGTTCTCTCGCCACTGGAAATC | ACGTTGGATGTCTCCTCAAAGAAGGAGGAC | GAAGGAGGACCCCTCAG |
| rs7421861 | ACGTTGGATGCTCTTCCTTCTACGTGAGGC | ACGTTGGATGGGTGAAGGCTCTTAGTAGGA | GGCTGCAGCTTCTGCACAGC |
| rs6710479 | ACGTTGGATGTCCACATAGGAATCTGTGGG | ACGTTGGATGTCTTCCAACTCCTCACAGTC | ACAGTCGTGTGTGTGTG |
| ***CTLA4*** |  |  |  |
| rs231775 | ACGTTGGATGCCTCCTCCATCTTCATGCTC | ACGTTGGATGCTGAACACCGCTCCCATAAA | CACAAGGCTCAGCTGAACCTGGCT |
| rs231777 | ACGTTGGATGACCTTATCTCTCTCTAGACC | ACGTTGGATGGAGTACCTACTTCATACAAAC | ATACAAACTACATGGTTTCTTA |
| rs231779 | ACGTTGGATGCACTCCCATGCTCCTTTGTT | ACGTTGGATGTGTCAAAGGGATTGAGCAGA | GCCACTATTTTTGAGTTGATGCAAG |
| ***HAVCR2*** |  |  |  |
| rs9313441 | ACGTTGGATGTGCCAGACACATAGTAGAGG | ACGTTGGATGTCAACGTGTGCCTTAGATAG | AGATAGTTATATGGACTGTGTTA |
| rs13170556 | ACGTTGGATGTTTGTGAATTGGACCATCCC | ACGTTGGATGAGTGTTGGCTACAAGGTGAC | TGACAGATCAAAGGTGATTTG |
| rs919744 | ACGTTGGATGTTCTTTCTGTGGCTGGAATC | ACGTTGGATGTTTGGGAACTGACACATTGG | GCTAGAATAAAGCCCATCA |
| rs1036199 | ACGTTGGATGCTGACATTAGCCAAGGTCAC | ACGTTGGATGCCTGGTGGTAAGCATCCTTG | CAGTGAAGTCTCTCTGC |
