# Supplementary material for: Association of tuberculosis risk with genetic polymorphisms of the immune checkpoint genes PDCD1, CTLA-4, and TIM3
Source: PLoS One. 2024 May 9;19(5):e0303431. doi: 10.1371/journal.pone.0303431 (PMC11081348; doi:10.1371/journal.pone.0303431)
Supplement: S3 Table — Abbreviations: df: degree of freedom. ars231777 TT genotype is only five subjects, none in female subjects and five in male subjects above 60 years old, so the df = 4. brs1036199 CC genotype, rs9313441 AA genotype, and rs919744 GG genotype are not detected in case and control groups, so the df = 3. cp value was calculated by logistic regression. (DOCX) [file pone.0303431.s003.docx]

**S3 Table. Interaction of genetic variation and sex/age contribute to tuberculosis risk.**

|  | ***χ*^2^** | **df** | ***p* value^c^** |
| --- | --- | --- | --- |
| **Sex*genotype** | | | |
| rs10204525 | 4.523 | 5 | 0.477 |
| rs2227982 | 5.725 | 5 | 0.334 |
| rs7421861 | 6.098 | 5 | 0.297 |
| rs6710479 | 8.201 | 5 | 0.145 |
| rs231775 | 5.542 | 5 | 0.353 |
| rs231777 | 4.330 | 4^a^ | 0.363 |
| rs231779 | 5.542 | 5 | 0.353 |
| rs9313441 | 3.091 | 3^b^ | 0.378 |
| rs13170556 | 13.165 | 5 | 0.022 |
| rs919744 | 5.001 | 3^b^ | 0.172 |
| rs1036199 | 5.001 | 3^b^ | 0.172 |
| **65 age*genotype** | | | |
| rs10204525 | 96.643 | 5 | <0.001 |
| rs2227982 | 99.361 | 5 | <0.001 |
| rs7421861 | 97.782 | 5 | <0.001 |
| rs6710479 | 99.169 | 5 | <0.001 |
| rs231775 | 101.745 | 5 | <0.001 |
| rs231777 | 96.944 | 4^a^ | <0.001 |
| rs231779 | 101.746 | 5 | <0.001 |
| rs9313441 | 95.745 | 3^b^ | <0.001 |
| rs13170556 | 98.024 | 5 | <0.001 |
| rs919744 | 97.198 | 3^b^ | <0.001 |
| rs1036199 | 97.198 | 3^b^ | <0.001 |

Abbreviations: df: degree of freedom.

^a^rs231777 TT genotype is only five subjects, none in female subjects and five in male subjects above 60 years old, so the df=4.

^b^rs1036199 CC genotype, rs9313441 AA genotype, and rs919744 GG genotype are not detected in case and control groups, so the df=3.

^c^*p* value was calculated by logistic regression.
