# Supplementary material for: Association of tuberculosis risk with genetic polymorphisms of the immune checkpoint genes PDCD1, CTLA-4, and TIM3
Source: PLoS One. 2024 May 9;19(5):e0303431. doi: 10.1371/journal.pone.0303431 (PMC11081348; doi:10.1371/journal.pone.0303431)
Supplement: S4 Table — Abbreviations: Ref., reference genotype; CI, confidence interval; OR, odds ratio; Pc, the Bonferroni correction of P values. aχ2 test. bAdj. = adjusted for sex by logistic regression. (DOCX) [file pone.0303431.s004.docx]

**S4 Table. The differences between groups with and without TB in genotypes and alleles frequencies of selected SNPs and results of odds ratio analysis in non-aged (<65-year-old) participants.**

| **SNP** | **Genotype** | **Counts** | | ***p* value^a^** | ***p_c_* value** | **Adj. OR (95% CI)^b^** | ***p* value for Adj. OR** |
| --- | --- | --- | --- | --- | --- | --- | --- |
|  |  | **TB group**  **n=184** | **Non-TB group**  **n=68** |  |  |  |  |
| ***PDCD1*** |  |  |  |  |  |  |  |
| rs10204525 | CC | 17 (9) | 6 (8) | 0.956 | NS | 1.148 (0.412, 3.202) | 0.792 |
|  | TC | 87 (47) | 31 (46) |  |  | 1.099 (0.612, 1.971) | 0.752 |
|  | TT (ref.) | 80 (44) | 31 (46) |  |  | 1 |  |
| Allele model | C | 121 (33) | 43 (32) | 0.788 | NS | 1.077 (0.705, 1.644) | 0.732 |
|  | T (ref.) | 247 (67) | 93 (68) |  |  | 1 |  |
| Dominant model | TT | 80 (44) | 31 (46) | 0.765 | NS | 0.904 (0.516, 1.584) | 0.724 |
|  | TC+CC (ref.) | 104 (56) | 37 (54) |  |  | 1 |  |
| Recessive model | CC | 17 (9) | 6 (8) | 0.919 | NS | 1.094 (0.410, 2.917) | 0.858 |
|  | TT+TC (ref.) | 167 (91) | 62 (92) |  |  | 1 |  |
| Overdominant model | TC | 87 (47) | 31 (46) | 0.811 | NS | 1.073 (0.613, 1.877) | 0.805 |
|  | TT+CC (ref.) | 97 (53) | 37 (54) |  |  | 1 |  |
| rs2227982 | AA | 38 (21) | 15 (22) | 0.695 | NS | 1.052 (0.485, 2.283) | 0.898 |
|  | GA | 92 (50) | 30 (44) |  |  | 1.320 (0.696, 2.505) | 0.395 |
|  | GG (ref.) | 54 (29) | 23 (34) |  |  | 1 |  |
| Allele model | A | 168 (46) | 60 (44) | 0.759 | NS | 1.053 (0.708, 1.566) | 0.797 |
|  | G (ref.) | 200 (54) | 76 (56) |  |  | 1 |  |
| Dominant model | GG | 54 (29) | 23 (34) | 0.494 | NS | 0.813 (0.448, 1.474) | 0.495 |
|  | AA+GA (ref.) | 130 (71) | 45 (66) |  |  | 1 |  |
| Recessive model | AA | 38 (21) | 15 (22) | 0.808 | NS | 0.892 (0.452, 1.760) | 0.741 |
|  | GA+GG (ref.) | 146 (79) | 53 (78) |  |  | 1 |  |
| Overdominant model | GA | 92 (50) | 30 (44) | 0.407 | NS | 1.294 (0.737, 2.270) | 0.369 |
|  | AA+GG (ref.) | 92 (50) | 38 (56) |  |  | 1 |  |
| rs7421861 | GG | 4 (2) | 0 | 0.472 | NS | ND | ND |
|  | GA | 56 (30) | 21 (31) |  |  | 1.043 (0.568, 1.916) | 0.893 |
|  | AA (ref.) | 124 (68) | 47 (69) |  |  | 1 |  |
| Allele model | G | 64 (17) | 21 (15) | 0.604 | NS | 1.185 (0.691, 2.035) | 0.537 |
|  | A (ref.) | 304 (83) | 115 (85) |  |  | 1 |  |
| Dominant model | AA | 124 (68) | 47 (69) | 0.795 | NS | 0.895 (0.489, 1.638) | 0.718 |
|  | GA+GG (ref.) | 60 (32) | 21 (31) |  |  | 1 |  |
| Recessive model | GG | 4 (2) | 0 | 0.220 | NS | ND | ND |
|  | AA+GA (ref.) | 180 (98) | 68 (100) |  |  |  |  |
| Overdominant model | GA | 56 (30) | 21 (31) | 0.945 | NS | 1.006 (0.548, 1.845) | 0.985 |
|  | AA+GG (ref.) | 128 (70) | 47 (69) |  |  | 1 |  |
| rs6710479 | CC | 17 (9) | 2 (3) | 0.242 | NS | 3.504 (0.766, 16.022) | 0.106 |
|  | CT | 72 (39) | 29 (43) |  |  | 0.998 (0.559, 1.781) | 0.995 |
|  | TT (ref.) | 95 (52) | 37 (54) |  |  | 1 |  |
| Allele model | C | 106 (29) | 33 (24) | 0.311 | NS | 1.298 (0.824, 2.047) | 0.261 |
|  | T (ref.) | 262 (71) | 103 (76) |  |  | 1 |  |
| Dominant model | TT | 95 (52) | 37 (54) | 0.695 | NS | 0.865 (0.493, 1.520) | 0.615 |
|  | CT+CC (ref.) | 89 (48) | 31 (46) |  |  | 1 |  |
| Recessive model | CC | 17 (9) | 2 (3) | 0.093 | NS | 3.507 (0.785, 15.672) | 0.100 |
|  | TT+CT (ref.) | 167 (91) | 66 (97) |  |  | 1 |  |
| Overdominant model | CT | 72 (39) | 29 (43) | 0.613 | NS | 0.882 (0.500, 1.556) | 0.666 |
|  | TT+CC (ref.) | 112 (61) | 39 (57) |  |  | 1 |  |
| ***CTLA4*** |  |  |  |  |  |  |  |
| rs231775 | AA | 25 (13) | 9 (13) | 0.238 | NS | 0.732 (0.295, 1.812) | 0.500 |
|  | AG | 82 (45) | 38 (56) |  |  | 0.594 (0.320, 1.103) | 0.099 |
|  | GG (ref.) | 77 (42) | 21 (31) |  |  | 1 |  |
| Allele model | A | 132 (36) | 56 (41) | 0.274 | NS | 0.790 (0.528, 1.183) | 0.253 |
|  | G (ref.) | 236 (64) | 80 (59) |  |  | 1 |  |
| Dominant model | GG | 77 (42) | 21 (31) | 0.113 | NS | 1.611 (0.890, 2.914) | 0.115 |
|  | AA+AG (ref.) | 107 (58) | 47 (69) |  |  | 1 |  |
| Recessive model | AA | 25 (13) | 9 (13) | 0.942 | NS | 0.987 (0.433, 2.253) | 0.975 |
|  | AG+GG (ref.) | 159 (87) | 59 (87) |  |  | 1 |  |
| Overdominant model | AG | 82 (45) | 38 (56) | 0.110 | NS | 0.646 (0.368, 1.133) | 0.128 |
|  | AA+GG (ref.) | 102 (55) | 30 (44) |  |  | 1 |  |
| rs231777 | TT | 0 | 1 (2) | 0.248 | NS | ND | ND |
|  | TC | 33 (18) | 13 (19) |  |  | 0.898 (0.439, 1.835) | 0.768 |
|  | CC (ref.) | 151 (82) | 54 (79) |  |  | 1 |  |
| Allele model | T | 33 (9) | 15 (11) | 0.484 | NS | 0.784 (0.411, 1.497) | 0.461 |
|  | C (ref.) | 335 (91) | 121 (89) |  |  | 1 |  |
| Dominant model | CC | 151 (82) | 54 (79) | 0.631 | NS | 1.201 (0.596, 2.418) | 0.608 |
|  | TT+TC (ref.) | 33 (18) | 14 (21) |  |  | 1 |  |
| Recessive model | TT | 0 | 1 (1) | 0.099 | NS | ND | ND |
|  | TC+CC (ref.) | 184 (100) | 67 (99) |  |  |  |  |
| Overdominant model | TC | 33 (18) | 13 (19) | 0.829 | NS | 0.916 (0.449, 1.870) | 0.809 |
|  | TT+CC (ref.) | 151 (82) | 55 (81) |  |  | 1 |  |
| rs231779 | CC | 25 (13) | 9 (13) | 0.238 | NS | 0.732 (0.295, 1.812) | 0.500 |
|  | CT | 82 (45) | 38 (56) |  |  | 0.594 (0.320, 1.103) | 0.099 |
|  | TT (ref.) | 77 (42) | 21 (31) |  |  | 1 |  |
| Allele model | C | 132 (36) | 56 (41) | 0.274 | NS | 0.790 (0.528, 1.183) | 0.253 |
|  | T (ref.) | 236 (64) | 80 (59) |  |  | 1 |  |
| Dominant model | TT | 77 (42) | 21 (31) | 0.113 | NS | 1.611 (0.890, 2.914) | 0.115 |
|  | CT+CC (ref.) | 107 (58) | 47 (69) |  |  | 1 |  |
| Recessive model | CC | 25 (13) | 9 (13) | 0.942 | NS | 0.987 (0.433, 2.253) | 0.975 |
|  | CT+TT (ref.) | 159 (87) | 59 (87) |  |  | 1 |  |
| Overdominant model | CT | 82 (45) | 38 (56) | 0.110 | NS | 0.646 (0.368, 1.133) | 0.128 |
|  | TT+CC (ref.) | 102 (55) | 30 (44) |  |  | 1 |  |
| ***HAVCR2*** |  |  |  |  |  |  |  |
| rs9313441 | AA | 0 | 0 |  |  | ND | ND |
|  | AG | 12 (6) | 7 (10) | 0.314 | NS | 0.598 (0.225, 1.593) | 0.304 |
|  | GG (ref.) | 172 (94) | 61 (90) |  |  | 1 |  |
| Allele model | A | 12 (3) | 7 (5) | 0.324 | NS | 0.612 (0.235, 1.591) | 0.314 |
|  | G (ref.) | 356 (97) | 129 (95) |  |  | 1 |  |
| Dominant model | GG | 172 (94) | 61 (90) | 0.314 | NS | 1.672 (0.628, 4.452) | 0.304 |
|  | AA+AG (ref.) | 12 (6) | 7 (10) |  |  | 1 |  |
| Recessive model | AA | 0 | 0 | ND | ND | ND | ND |
|  | AG+GG (ref.) | 184 (100) | 68 (100) |  |  |  |  |
| Overdominant model | AG | 12 (6) | 7 (10) | 0.314 | NS | 0.598 (0.225, 1.593) | 0.304 |
|  | AA+GG (ref.) | 172 (94) | 61 (90) |  |  | 1 |  |
| rs13170556 | CC | 10 (6) | 1 (1) | 0.200 | NS | 4.529 (0.561, 36.543) | 0.156 |
|  | TC | 63 (34) | 19 (28) |  |  | 1.421 (0.768, 2.631) | 0.263 |
|  | TT (ref.) | 111 (60) | 48 (71) |  |  | 1 |  |
| Allele model | C | 83 (23) | 21 (15) | 0.080 | NS | 1.602 (0.946, 2.711) | 0.079 |
|  | T (ref.) | 285 (77) | 115 (85) |  |  | 1 |  |
| Dominant model | TT | 111 (60) | 48 (71) | 0.134 | NS | 0.635 (0.348, 1.157) | 0.138 |
|  | TC+CC (ref.) | 73 (40) | 20 (29) |  |  | 1 |  |
| Recessive model | CC | 10 (6) | 1 (1) | 0.172 | NS | 4.053 (0.506, 32.439) | 0.187 |
|  | TC+TT (ref.) | 174 (94) | 67 (99) |  |  | 1 |  |
| Overdominant model | TC | 63 (34) | 19 (28) | 0.344 | NS | 1.329 (0.720, 2.451) | 0.363 |
|  | TT+CC (ref.) | 121 (66) | 49 (72) |  |  | 1 |  |
| rs919744 | GG | 0 | 0 |  |  | ND | ND |
|  | GC | 8 (4) | 1 (1) | 0.275 | NS | 2.893 (0.354, 23.681) | 0.322 |
|  | CC (ref.) | 176 (96) | 67 (99) |  |  | 1 |  |
| Allele model | G | 8 (2) | 1 (1) | 0.279 | NS | 2.845 (0.352, 23.021) | 0.327 |
|  | C (ref.) | 360 (98) | 135 (99) |  |  | 1 |  |
| Dominant model | CC | 176 (96) | 67 (99) | 0.275 | NS | 0.346 (0.042, 2.829) | 0.322 |
|  | GC+GG (ref.) | 8 (4) | 1 (1) |  |  | 1 |  |
| Recessive model | GG | 0 | 0 | ND | ND | ND | ND |
|  | GC+CC (ref.) | 184 (100) | 68 (100) |  |  |  |  |
| Overdominant model | GC | 8 (4) | 1 (1) | 0.275 | NS | 2.893 (0.354, 23.681) | 0.322 |
|  | GG+CC (ref.) | 176 (96) | 67 (99) |  |  | 1 |  |
| rs1036199 | CC | 0 | 0 |  |  | ND | ND |
|  | CA | 8 (4) | 1 (1) | 0.275 | NS | 2.893 (0.354, 23.681) | 0.322 |
|  | AA (ref.) | 176 (96) | 67 (99) |  |  | 1 |  |
| Allele model | C | 8 (2) | 1 (1) | 0.279 | NS | 2.845 (0.352, 23.021) | 0.327 |
|  | A (ref.) | 360 (98) | 135 (99) |  |  | 1 |  |
| Dominant model | AA | 176 (96) | 67 (99) | 0.275 | NS | 0.346 (0.042, 2.829) | 0.322 |
|  | CA+CC (ref.) | 8 (4) | 1 (1) |  |  | 1 |  |
| Recessive model | CC | 0 | 0 | ND | ND | ND | ND |
|  | CA+AA (ref.) | 184 (100) | 68 (100) |  |  |  |  |
| Overdominant model | CA | 8 (4) | 1 (1) | 0.275 | NS | 2.893 (0.354, 23.681) | 0.322 |
|  | AA+CC (ref.) | 176 (96) | 67 (99) |  |  | 1 |  |

Abbreviations: Ref., reference genotype; CI, confidence interval; OR, odds ratio; Pc, the Bonferroni correction of P values.

^a^*χ*^2^ test.

^b^Adj. = adjusted for sex by logistic regression.
