# Supplementary material for: Association of tuberculosis risk with genetic polymorphisms of the immune checkpoint genes PDCD1, CTLA-4, and TIM3
Source: PLoS One. 2024 May 9;19(5):e0303431. doi: 10.1371/journal.pone.0303431 (PMC11081348; doi:10.1371/journal.pone.0303431)
Supplement: S5 Table — Abbreviations: Ref., reference genotype; CI, confidence interval; OR, odds ratio; Pc, the Bonferroni correction of P values. aχ2 test. bAdj. = adjusted for sex by logistic regression. (DOCX) [file pone.0303431.s005.docx]

**S5 Table. The differences between groups with and without TB in genotypes and alleles frequencies of selected SNPs and results of odds ratio analysis in aged (≥65-year-old) participants.**

| **SNP** | **Genotype** | **Counts** | | ***p* value^a^** | ***p_c_* value** | **Adj. OR (95% CI)^b^** | ***p* value for Adj. OR** |
| --- | --- | --- | --- | --- | --- | --- | --- |
|  |  | **TB group**  **n=101** | **Non-TB group**  **n=202** |  |  |  |  |
| ***PDCD1*** |  |  |  |  |  |  |  |
| rs10204525 | CC | 8 (8) | 18 (9) | 0.512 | NS | 1.013 (0.411, 2.496) | 0.978 |
|  | TC | 46 (45) | 78 (39) |  |  | 1.339 (0.811, 2.212) | 0.254 |
|  | TT (ref.) | 47 (47) | 106 (52) |  |  | 1 |  |
| Allele model | C | 62 (31) | 114 (28) | 0.527 | NS | 1.133 (0.783, 1.640) | 0.509 |
|  | T (ref.) | 140 (69) | 290 (72) |  |  | 1 |  |
| Dominant model | TT | 47 (47) | 106 (52) | 0.330 | NS | 0.782 (0.484, 1.264) | 0.316 |
|  | TC+CC (ref.) | 54 (53) | 96 (48) |  |  | 1 |  |
| Recessive model | CC | 8 (8) | 18 (9) | 0.772 | NS | 0.885 (0.371, 2.112) | 0.783 |
|  | TT+TC (ref.) | 93 (92) | 184 (91) |  |  | 1 |  |
| Overdominant model | TC | 46 (45) | 78 (39) | 0.247 | NS | 1.337 (0.824, 2.169) | 0.240 |
|  | TT+CC (ref.) | 55 (55) | 124 (61) |  |  | 1 |  |
| rs2227982 | AA | 23 (23) | 59 (29) | 0.347 | NS | 0.834 (0.417, 1.669) | 0.608 |
|  | GA | 55 (54) | 93 (46) |  |  | 1.272 (0.700, 2.313) | 0.430 |
|  | GG (ref.) | 23 (23) | 50 (25) |  |  | 1 |  |
| Allele model | A | 101 (50) | 211 (52) | 0.605 | NS | 0.907 (0.646, 1.273) | 0.574 |
|  | G (ref.) | 101 (50) | 193 (48) |  |  | 1 |  |
| Dominant model | GG | 23 (23) | 50 (25) | 0.704 | NS | 0.906 (0.514, 1.597) | 0.734 |
|  | AA+GA (ref.) | 78 (77) | 152 (75) |  |  | 1 |  |
| Recessive model | AA | 23 (23) | 59 (29) | 0.235 | NS | 0.707 (0.405, 1.235) | 0.223 |
|  | GA+GG (ref.) | 78 (77) | 143 (71) |  |  | 1 |  |
| Overdominant model | GA | 55 (54) | 93 (46) | 0.167 | NS | 1.399 (0.866, 2.260) | 0.170 |
|  | AA+GG (ref.) | 46 (46) | 109 (54) |  |  | 1 |  |
| rs7421861 | GG | 2 (2) | 8 (4) | 0.465 | NS | 0.533 (0.110, 2.584) | 0.434 |
|  | GA | 29 (29) | 48 (24) |  |  | 1.261 (0.733, 2.169) | 0.401 |
|  | AA (ref.) | 70 (69) | 146 (72) |  |  | 1 |  |
| Allele model | G | 33 (16) | 64 (16) | 0.875 | NS | 1.043 (0.659, 1.651) | 0.856 |
|  | A (ref.) | 169 (84) | 340 (84) |  |  | 1 |  |
| Dominant model | AA | 70 (69) | 146 (72) | 0.590 | NS | 0.863 (0.511, 1.456) | 0.581 |
|  | GA+GG (ref.) | 31 (31) | 56 (28) |  |  | 1 |  |
| Recessive model | GG | 2 (2) | 8 (4) | 0.363 | NS | 0.500 (0.104, 2.408) | 0.387 |
|  | AA+GA (ref.) | 99 (98) | 194 (96) |  |  | 1 |  |
| Overdominant model | GA | 29 (29) | 48 (24) | 0.351 | NS | 1.292 (0.753, 2.216) | 0.352 |
|  | AA+GG (ref.) | 72 (71) | 154 (76) |  |  | 1 |  |
| rs6710479 | CC | 3 (3) | 12 (6) | 0.172 | NS | 0.575 (0.156, 2.125) | 0.406 |
|  | CT | 44 (44) | 68 (34) |  |  | 1.472 (0.895, 2.421) | 0.128 |
|  | TT (ref.) | 54 (53) | 122 (60) |  |  | 1 |  |
| Allele model | C | 50 (25) | 92 (23) | 0.587 | NS | 1.124 (0.756, 1.670) | 0.563 |
|  | T (ref.) | 152 (75) | 312 (77) |  |  | 1 |  |
| Dominant model | TT | 54 (53) | 122 (60) | 0.249 | NS | 0.746 (0.460, 1.211) | 0.236 |
|  | CT+CC (ref.) | 47 (47) | 80 (40) |  |  | 1 |  |
| Recessive model | CC | 3 (3) | 12 (6) | 0.261 | NS | 0.491 (0.135, 1.782) | 0.279 |
|  | TT+CT (ref.) | 98 (97) | 190 (94) |  |  | 1 |  |
| Overdominant model | CT | 44 (44) | 68 (34) | 0.092 | NS | 1.531 (0.937, 2.501) | 0.089 |
|  | TT+CC (ref.) | 57 (56) | 134 (66) |  |  | 1 |  |
| ***CTLA4*** |  |  |  |  |  |  |  |
| rs231775 | AA | 6 (6) | 20 (10) | 0.426 | NS | 0.536 (0.202, 1.426) | 0.212 |
|  | AG | 46 (45) | 95 (47) |  |  | 0.862 (0.524, 1.417) | 0.558 |
|  | GG (ref.) | 49 (49) | 87 (43) |  |  | 1 |  |
| Allele model | A | 58 (29) | 135 (33) | 0.241 | NS | 0.805 (0.557, 1.164) | 0.249 |
|  | G (ref.) | 144 (71) | 269 (67) |  |  | 1 |  |
| Dominant model | GG | 49 (49) | 87 (43) | 0.369 | NS | 1.241 (0.768, 2.006) | 0.377 |
|  | AA+AG (ref.) | 52 (51) | 115 (57) |  |  | 1 |  |
| Recessive model | AA | 6 (6) | 20 (10) | 0.246 | NS | 0.578 (0.224, 1.489) | 0.256 |
|  | AG+GG (ref.) | 95 (94) | 182 (90) |  |  | 1 |  |
| Overdominant model | AG | 46 (45) | 95 (47) | 0.807 | NS | 0.944 (0.584, 1.524) | 0.812 |
|  | AA+GG (ref.) | 55 (55) | 107 (53) |  |  | 1 |  |
| rs231777 | TT | 1 (1) | 3 (1) | 0.426 | NS | 0.733 (0.075, 7.178) | 0.790 |
|  | TC | 22 (22) | 32 (16) |  |  | 1.480 (0.807, 2.715) | 0.205 |
|  | CC (ref.) | 78 (77) | 167 (83) |  |  | 1 |  |
| Allele model | T | 24 (12) | 38 (9) | 0.343 | NS | 1.309 (0.761, 2.251) | 0.331 |
|  | C (ref.) | 178 (88) | 366 (91) |  |  | 1 |  |
| Dominant model | CC | 78 (77) | 167 (83) | 0.256 | NS | 0.705 (0.390, 1.275) | 0.248 |
|  | TT+TC (ref.) | 23 (23) | 35 (17) |  |  | 1 |  |
| Recessive model | TT | 1 (1) | 3 (1) | 0.722 | NS | 0.679 (0.070, 6.625) | 0.739 |
|  | TC+CC (ref.) | 100 (99) | 199 (99) |  |  | 1 |  |
| Overdominant model | TC | 22 (22) | 32 (16) | 0.203 | NS | 1.488 (0.812, 2.726) | 0.199 |
|  | TT+CC (ref.) | 79 (78) | 170 (84) |  |  | 1 |  |
| rs231779 | CC | 6 (6) | 20 (10) | 0.426 | NS | 0.536 (0.202, 1.426) | 0.212 |
|  | CT | 46 (45) | 95 (47) |  |  | 0.862 (0.524, 1.417) | 0.558 |
|  | TT (ref.) | 49 (49) | 87 (43) |  |  | 1 |  |
| Allele model | C | 58 (29) | 135 (33) | 0.241 | NS | 0.805 (0.557, 1.164) | 0.249 |
|  | T (ref.) | 144 (71) | 269 (67) |  |  | 1 |  |
| Dominant model | TT | 49 (49) | 87 (43) | 0.369 | NS | 1.241 (0.768, 2.006) | 0.377 |
|  | CT+CC (ref.) | 52 (51) | 115 (57) |  |  | 1 |  |
| Recessive model | CC | 6 (6) | 20 (10) | 0.246 | NS | 0.578 (0.224, 1.489) | 0.256 |
|  | CT+TT (ref.) | 95 (94) | 182 (90) |  |  | 1 |  |
| Overdominant model | CT | 46 (45) | 95 (47) | 0.807 | NS | 0.944 (0.584, 1.524) | 0.812 |
|  | TT+CC (ref.) | 55 (55) | 107 (53) |  |  | 1 |  |
| ***HAVCR2*** |  |  |  |  |  |  |  |
| rs9313441 | AA | 0 | 0 |  |  | ND | ND |
|  | AG | 10 (10) | 16 (8) | 0.562 | NS | 1.246 (0.537, 2.887) | 0.609 |
|  | GG (ref.) | 91 (90) | 186 (92) |  |  | 1 |  |
| Allele model | A | 10 (5) | 16 (4) | 0.571 | NS | 1.229 (0.544, 2.776) | 0.619 |
|  | G (ref.) | 192 (95) | 388 (96) |  |  | 1 |  |
| Dominant model | GG | 91 (90) | 186 (92) | 0.562 | NS | 0.803 (0.346, 1.861) | 0.609 |
|  | AA+AG (ref.) | 10 (10) | 16 (8) |  |  | 1 |  |
| Recessive model | AA | 0 | 0 | ND | ND | ND | ND |
|  | AG+GG (ref.) | 101 (100) | 202 (100) |  |  |  |  |
| Overdominant model | AG | 10 (10) | 16 (8) | 0.562 | NS | 1.246 (0.537, 2.887) | 0.609 |
|  | AA+GG (ref.) | 91 (90) | 186 (92) |  |  | 1 |  |
| rs13170556 | CC | 3 (3) | 7 (3) | 0.634 | NS | 0.891 (0.223, 3.565) | 0.870 |
|  | TC | 28 (28) | 46 (23) |  |  | 1.293 (0.746, 2.239) | 0.360 |
|  | TT (ref.) | 70 (69) | 149 (74) |  |  | 1 |  |
| Allele model | C | 34 (17) | 60 (15) | 0.526 | NS | 1.153 (0.728, 1.827) | 0.544 |
|  | T (ref.) | 168 (83) | 344 (85) |  |  | 1 |  |
| Dominant model | TT | 70 (69) | 149 (74) | 0.414 | NS | 0.807 (0.476, 1.366) | 0.424 |
|  | TC+CC (ref.) | 31 (31) | 53 (26) |  |  | 1 |  |
| Recessive model | CC | 3 (3) | 7 (3) | 0.820 | NS | 0.833 (0.210, 3.305) | 0.795 |
|  | TC+TT (ref.) | 98 (97) | 195 (97) |  |  | 1 |  |
| Overdominant model | TC | 28 (28) | 46 (23) | 0.344 | NS | 1.299 (0.753, 2.243) | 0.347 |
|  | TT+CC (ref.) | 73 (72) | 156 (77) |  |  | 1 |  |
| rs919744 | GG | 0 | 0 |  |  | ND | ND |
|  | GC | 1 (1) | 6 (3) | 0.279 | NS | 0.309 (0.036, 2.626) | 0.282 |
|  | CC (ref.) | 100 (99) | 196 (97) |  |  | 1 |  |
| Allele model | G | 1 (1) | 6 (1) | 0.282 | NS | 0.314 (0.037, 2.641) | 0.286 |
|  | C (ref.) | 201 (99) | 398 (99) |  |  | 1 |  |
| Dominant model | CC | 100 (99) | 196 (97) | 0.279 | NS | 3.241 (0.381, 27.585) | 0.282 |
|  | GC+GG (ref.) | 1 (1) | 6 (3) |  |  | 1 |  |
| Recessive model | GG | 0 | 0 | ND | ND | ND | ND |
|  | GC+CC (ref.) | 101 (100) | 202 (100) |  |  |  |  |
| Overdominant model | GC | 1 (1) | 6 (3) | 0.279 | NS | 0.309 (0.036, 2.626) | 0.282 |
|  | GG+CC (ref.) | 100 (99) | 196 (97) |  |  | 1 |  |
| rs1036199 | CC | 0 | 0 |  |  | ND | ND |
|  | CA | 1 (1) | 6 (3) | 0.279 | NS | 0.309 (0.036, 2.626) | 0.282 |
|  | AA (ref.) | 100 (99) | 196 (97) |  |  | 1 |  |
| Allele model | C | 1 (1) | 6 (1) | 0.282 | NS | 0.314 (0.037, 2.641) | 0.286 |
|  | A (ref.) | 201 (99) | 398 (99) |  |  | 1 |  |
| Dominant model | AA | 100 (99) | 196 (97) | 0.279 | NS | 3.241 (0.381, 27.585) | 0.282 |
|  | CA+CC (ref.) | 1 (1) | 6 (3) |  |  | 1 |  |
| Recessive model | CC | 0 | 0 | ND | ND | ND | ND |
|  | CA+AA (ref.) | 101 (100) | 202 (100) |  |  |  |  |
| Overdominant model | CA | 1 (1) | 6 (3) | 0.279 | NS | 0.309 (0.036, 2.626) | 0.282 |
|  | AA+CC (ref.) | 100 (99) | 196 (97) |  |  | 1 |  |

Abbreviations: Ref., reference genotype; CI, confidence interval; OR, odds ratio; Pc, the Bonferroni correction of P values.

^a^*χ*^2^ test.

^b^Adj. = adjusted for sex by logistic regression.
