# Supplementary material for: Association of tuberculosis risk with genetic polymorphisms of the immune checkpoint genes PDCD1, CTLA-4, and TIM3
Source: PLoS One. 2024 May 9;19(5):e0303431. doi: 10.1371/journal.pone.0303431 (PMC11081348; doi:10.1371/journal.pone.0303431)
Supplement: S6 Table — Abbreviations: Ref., reference genotype; CI, confidence interval; OR, odds ratio; Pc, the Bonferroni correction of P values. aχ2 test. bAdj. = adjusted for age and sex by logistic regression. (DOCX) [file pone.0303431.s006.docx]

**S6 Table. Haplotype distribution of the two investigated *PDCD1* and *CTLA4* polymorphisms in the age-stratified populations.**

| **Haplotype** | | **Frequency (%)** | **TB (n)** | **Non-TB (n)** | ***p* value^a^** | ***p_c_* value** | **Adj. OR (95% CI)^b^** | ***p* value for OR** |
| --- | --- | --- | --- | --- | --- | --- | --- | --- |
| **rs2227982-rs7421861-rs6710479** | |  |  |  |  |  |  |  |
| < 65 | A-A-T (ref.) | 44.6 | 165 | 60 | 0.359 | NS | 1.000 |  |
|  | G-A-T | 26.8 | 92 | 43 |  |  | 0.775 (0.485, 1.238) | 0.286 |
|  | G-G-C | 15.9 | 59 | 21 |  |  | 1.055 (0.589, 1.888) | 0.858 |
|  | G-A-C | 11.1 | 44 | 12 |  |  | 1.351 (0.668, 2.733) | 0.403 |
|  | G-G-T | 1.0 | 5 | 0 |  |  | ND | ND |
|  | A-A-C | 0.6 | 3 | 0 |  |  | ND | ND |
| ≥ 65 | A-A-T (ref.) | 51.5 | 101 | 211 | 0.924 | NS | 1.000 |  |
|  | G-A-T | 24.7 | 50 | 100 |  |  | 1.052 (0.695, 1.593) | 0.810 |
|  | G-G-C | 15.7 | 32 | 63 |  |  | 1.072 (0.658, 1.746) | 0.781 |
|  | G-A-C | 7.8 | 18 | 29 |  |  | 1.307 (0.693, 2.467) | 0.408 |
|  | G-G-T | 0.3 | 1 | 1 |  |  | 1.991 (0.123, 32.336) | 0.628 |
|  | A-A-C | 0 | 0 | 0 |  |  | ND | ND |
| **rs231775-rs231777-rs231779** | |  |  |  |  |  |  |  |
| < 65 | G-C-T (ref.) | 62.7 | 236 | 80 | 0.636 | NS | 1.000 |  |
|  | A-C-C | 27.6 | 98 | 41 |  |  | 0.801 (0.513, 1.250) | 0.329 |
|  | A-T-C | 9.5 | 33 | 15 |  |  | 0.733 (0.378, 1.423) | 0.733 |
|  | G-C-C | 0.2 | 1 | 0 |  |  | ND | ND |
| ≥ 65 | G-C-T (ref.) | 68.2 | 144 | 269 | 0.108 | NS | 1.000 |  |
|  | A-C-C | 21.6 | 34 | 97 |  |  | 0.656 (0.422, 1.018) | 0.060 |
|  | A-T-C | 10.2 | 24 | 38 |  |  | 1.189 (0.686, 2.062) | 0.537 |
|  | G-C-C | 0 | 0 | 0 |  |  | ND | ND |

Abbreviations: Ref., reference genotype; CI, confidence interval; OR, odds ratio; Pc, the Bonferroni correction of P values.

^a^*χ*^2^ test.

^b^Adj. = adjusted for age and sex by logistic regression.
